# Introns Structure Patterns of Variation in Nucleotide Composition in *Arabidopsis thaliana* and Rice Protein-Coding Genes

**DOI:** 10.1093/gbe/evv189

**Published:** 2015-10-07

**Authors:** Adrienne Ressayre, Sylvain Glémin, Pierre Montalent, Laurana Serre-Giardi, Christine Dillmann, Johann Joets

**Affiliations:** ^1^UMR 0320/UMR 8120 Génétique Quantitative et Evolution—Le Moulon, INRA, Gif-sur-Yvette, France; ^2^Institut des Sciences de l’Evolution (ISEM), UMR 5554, Université de Montpellier, CNRS-IRD-EPHE, France; ^3^Department of Ecology and Genetics, Evolutionary Biology Centre, Uppsala University, Sweden; ^4^UMR 1345 IRHS Institut de Recherche en Horticulture et Semences, INRA, Centre de Recherche Angers-Nantes, Beaucousé, France; ^5^UMR 0320/UMR 8120 Génétique Quantitative et Evolution—Le Moulon, Université Paris-Sud, Gif-sur-Yvette, France

**Keywords:** intron, nucleotide composition, protein-coding genes, *Arabidopsis thaliana*, *Oryza sativa*

## Abstract

Plant genomes present a continuous range of variation in nucleotide composition (*G* + *C* content). In coding regions, *G* + *C*-poor species tend to have unimodal distributions of *G* + *C* content among genes within genomes and slight 5′–3′ gradients along genes. In contrast, *G* + *C*-rich species display bimodal distributions of *G* + *C* content among genes and steep 5′–3′ decreasing gradients along genes. The causes of these peculiar patterns are still poorly understood. Within two species (*Arabidopsis thaliana* and rice), each representative of one side of the continuum, we studied the consequences of intron presence on coding region and intron *G* + *C* content at different scales. By properly taking intron structure into account, we showed that, in both species, intron presence is associated with step changes in nucleotide, codon, and amino acid composition. This suggests that introns have a barrier effect structuring *G* + *C* content along genes and that previous continuous characterizations of the 5′–3′ gradients were artifactual. In external gene regions (located upstream first or downstream last introns), species-specific factors, such as GC-biased gene conversion, are shaping *G* + *C* content whereas in internal gene regions (surrounded by introns), *G* + *C* content is likely constrained to remain within a range common to both species.

## Introduction

In plants, average coding sequence (CDS) nucleotide composition (*G* + *C* content hereafter denoted GC-content) is highly variable between species and ranges from 40% to 60% (Serres-Giardi et al. 2012). Plant genomes also host more than 100,000 introns within their genes that occupy around one-quarter of the genic space. Like in other eukaryotes, plant introns tend to be GC-poor as compared with coding regions, and differences between both kinds of regions seem to be related with splicing efficiency (Goodall and Filipowicz 1989, 1991; Carle-Urioste et al. 1997; Carels and Bernardi 2000; Zhu et al. 2009; Amit et al. 2012). The intron–exon architecture also overlaps with chromatin organization, nucleosomes preferentially occupying exons whereas linkers are mainly formed by introns (Andersson et al. 2009; Chodavarapu et al. 2010; Amit et al. 2012). Nucleosome occupancy itself is principally determined by sequence GC-content (Tillo and Hughes 2009). As a consequence, the alternation of noncoding and coding sequences coincides in plants with a mosaic of GC-poor and GC-rich regions which corresponds to different chromatin domains.

Introns or splicing is implicated into a wide range of critical processes regarding gene expression (Lynch 2002; Maniatis and Reed 2002; Moore and Proudfoot 2009; Carmel and Chorev 2012). Splicing processes affect gene expression from transcription initiation and 5′-capping to poly-adenylation, export from the nucleus and even to the first round of translation. All these functional aspects are associated with a range of selective pressures unrelated to protein sequence that indirectly affects molecular rates of protein evolution (Warnecke et al. 2009; Shabalina et al. 2013; Weatheritt and Babu 2013). For example, correct splicing or alternative splicing is required for the production of functional transcript. Hence, canonical splicing motifs (5′ and 3′ splice sites) as well as enhancer motifs located near splice junctions are under selection and affect codon usage in neighboring CDSs in a variety of eukaryotes (Comeron and Guthrie 2005; Parmley et al. 2007; Warnecke and Hurst 2007; Ke et al. 2008; Larracuente et al. 2008; Denisov et al. 2014; Falanga et al. 2014). Furthermore, variation in intron number, intron location, and/or intron length are recurrently reported as associated with differences in GC-content of both introns and exons (Carels and Bernardi 2000; Wang et al. 2004; Guo et al. 2007; Zhu et al. 2009; Amit et al. 2012; Clément et al. 2015).

Whereas GC-poor plant genomes tend to display low level of variation among genes, the increase in genome-wide GC-content is associated with an increase in GC-content variability among genes within genomes leading to a bimodal distribution of CDS GC-content in GC-rich genomes. Within plant genomes, coding region GC-content also varies along genes, following a 5′–3′ decreasing GC gradient which steepness increases with genome-wide GC-content (Wong et al. 2002; Serres-Giardi et al. 2012; Clément et al. 2015). Indeed, part of the variation in GC-content between genes could be explained by variations in the gradient amplitude associated with the variation in gene length (Wong et al. 2002; Glémin et al. 2014).

Recently, two studies in plants revealed the existence of a close association between patterns of variation in GC-content in coding regions, introns, and recombination rates (Choi et al. 2013; Hellsten et al. 2013). This association provides a potential mechanism to explain how the intron–exon architecture of genes can influence nucleotide composition through GC-biased gene conversion (gBGC) (Duret and Galtier 2009; Glémin et al. 2014). gBGC is a process associated with recombination in several eukaryotes that favors the transmission of G and C alleles at meiosis (Duret and Galtier 2009). Introns could contribute to the formation of GC-content gradients either indirectly, because they take downstream CDSs away from the places where gBGC occurs, or directly, for example, by disrupting conversion tract. However, it is not clear how gBGC may interact or not with potential selective constraints on exons versus introns GC-content. In plant genes, three intermingled levels of structuring are described: 1) Systematic differences between introns and coding regions (Goodall and Filipowicz 1989, 1991; Carle-Urioste et al. 1997), 2) systematic differences between codon position within coding regions (Wong et al. 2002; Shi et al. 2006), and 3) decreasing 5′ to 3′ GC gradients of varying amplitudes (Wong et al. 2002; Zhu et al. 2009; Serres-Giardi et al. 2012). Indeed, the complex patterns of variation in GC-content along plant genes have prevented a clear understanding of the evolutionary forces acting on it. Moreover, until now, these patterns were usually described without taking into account gene intron number or precise gene architecture.

To investigate the potential role of introns in GC-content variation in plant genes, we reanalyzed the genomic data of *Arabidopsis thaliana* and rice (*Oryza sativa*), two species each representative of GC-poor or GC-rich plant genomes and offering the best annotated gene structures among plants. Compared with the GC-poor genome of *A. thaliana*, rice genome is characterized by a sharp genome-wide increase in gene GC-content (Carels and Bernardi 2000; Serres-Giardi et al. 2012). We focused on the link between intron presence and GC-gradients, analyzing patterns of GC-content variation at different scales from the nucleotide level to the gene scale within each of the two genomes. Within both genomes, we observed tight links between intron presence and variation in GC-content at sequence level that were translated at gene scale into a negative correlation between intron number and GC-content. Comparisons between those widely divergent genomes revealed that most of the differences in GC-content are concentrated in external gene regions, upstream first or downstream last intron. In contrast, central gene regions, surrounded by introns, are strikingly similar suggesting that introns have a barrier effect in both species, confining the GC-content increase of the rice genome into gene external regions. In addition, negative correlations among codon positions inside internal coding regions suggest stabilizing selection for a GC-content level that was similar in both species. Finally, the intron/exon architecture was shown to affect all codon positions and reveals a pervasive impact of gene intron–exon architecture on nucleotide, codon, and amino acid compositions.

## Materials and Methods

### Genomic Data

**Fig. 1.—:**
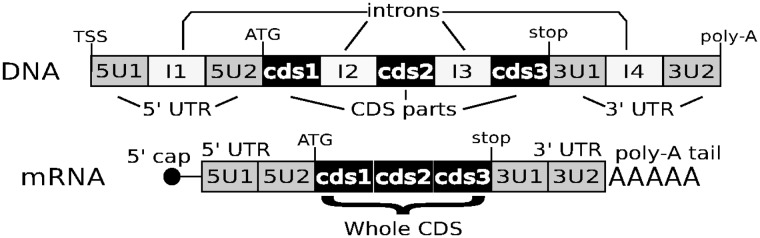
Protein-coding gene structure. A gene is composed of a variable number of regions that can be of four types: 5′-UTR, coding regions (cds), intron, and 3′-UTR. Introns are noncoding regions interrupting any of the other types of regions. They are excised and the remaining regions are spliced to produce the mature mRNA. 5′- and 3′-UTRs occupy 5′ and 3′ gene and mRNA extremities. CDSs encode for the amino acid sequence of the protein. Along a gene, each region was denoted according to its type and its rank position from the 5′-end toward the 3′-end. In the example shown in the figure, the gene has four introns, one intron in each UTRs and two introns in the coding region. Its decomposition in elements is provided by the list (5U1, I1, 5U2, cds1, I2, cds2, I3, cds3, 3U1, I4, 3U2), the whole CDS consisting in the three concatenated CDS parts (cds1cds2cds3). CDS will be used to refer to the complete CDS of a gene regardless of whether it is interrupted or not by introns. TSS is for transcription start site.

Genomic data come from whole-genome annotations of *A. thaliana* and *O. sativa* var. *nipponbare*. Exons are classically defined as the parts of the nucleotide (nt) sequence of genes that are assembled into mRNA sequence, introns being removed during or soon after transcription. However, they can be further divided into 5′-untranslated region (UTR), coding and 3′-UTR (see fig. 1). Our preliminary analyses (data not shown) indicated 1) significant differences in average GC-content occurred between coding and UTR, and 2) significant but low correlations between coding and UTR. For these reasons, we decided to study all four types of regions separately (5′-UTR, CDS, intron, and 3′-UTR) and to use the term CDS part instead of exon for coding region pieces to avoid confusion. Along a gene, each region was denoted according to its type and numbered according to its rank from the 5′-end toward the 3′-end (fig. 1).

#### Arabidopsis thaliana

We used the TAIR10 genome release (ftp://ftp.arabidopsis.org/). A single gene model was collected per locus/protein-coding gene: The one having the longest CDS and the highest confidence score. For each of the selected models we recorded the confidence score, chromosome number, gene start and stop, number of introns, as well as start and stop position of each of the different elements composing the gene. Only genes with a confidence score higher or equal to 4 were conserved.

#### Oryza sativa

We used the Release 6.1 of the MSU Rice Genome Annotation Project (ftp://ftp.plantbiology.msu.edu). No confidence score similar to the one existing in *Arabidopsis* is provided. However, when expression data such as ESTs (expressed sequence tags) or Full Length cDNA (FLcDNA) are available, it is indicated. We kept only gene models supported by full length cDNA. The same data as for *A. thaliana* were recorded for each selected gene model.

#### Final Data Sets

We restricted our analyses to gene models with both UTRs described and supported by expression data, ending up with a similar number of genes in both species (18,134 in *A. thaliana* and 17,862 in rice). Gene models for which at least one element was less than 10 nt long were discarded to avoid to compute GC-content on too few nts. In the two studied species, most of the genes do not have intron inserted in UTRs (around 79% in *A. thaliana* and 75% in rice). For those genes, the distribution of intron number within coding regions was similar between species (supplementary table S1, Supplementary Material online) and we decided to use these gene sets as reference. Two additional data sets were formed within each species, a first one made of the genes having any number of introns inserted within their coding regions plus a single intron inserted within their 5′-UTR (5′-UTR sets), and a second containing genes having any number of introns inserted within their coding regions plus a single intron inserted within the 3′-UTR (3′-UTR sets). Although the 5′-UTR data sets comprise roughly the same number of genes in the two species, intron presence in 3′-UTRs in rice is more than twice as frequent as in *A. thaliana* (supplementary table S1, Supplementary Material online). In both species, the remaining set of genes (4,024 in *A. thaliana* and 4,438 in rice) displays a high number of different combinations of intron insertion within UTRs and was discarded. Finally, to ensure that intron number classes comprise in general more than 100 genes for the reference data sets and the 5′-UTR data sets, we restricted our analyses to genes with less than 15 introns in the two reference sets and less than 9 introns in the UTR’s data sets. We ended up with six data sets which composition is shown in supplementary table S1, Supplementary Material online. To further study the consequences of the insertion of introns within UTRs, we also retrieved the paralogous set of genes available on the MSU site at http://rice.plantbiology.msu.edu/annotation_pseudo_ortho.shtml. This data set contains for both species the genes that are orthologous within a genome (duplicated genes) and we used it to produce in each species an additional data set composed of duplicated genes that differ by the presence/absence of a single additional intron inserted in the either 5′-UTR or 3′-UTR (supplementary table S2, Supplementary Material online).

### Compositional Analyses

#### GC-Content Measurements

In each species, genes were distributed into classes according to their intron number. Let *i* be the intron number of the genes and *N_i_* the number of genes with *i* introns (i∈{0,…,14}). Within classes, genes were denoted by the indices *g* ($g\in \{1,\ldots ,N_{i}\}$) and CDS parts by the indices j (j∈{1,…,i+1}). In both species, for each of the selected gene model, gene sequences were retrieved and analyzed using R and the bioconductor package (Gentleman et al. 2004; R Core Team 2013). After sorting genes according to intron number, we explored GC-content pattern of variation at different scales.

To study transitions between coding regions and introns, we computed GC-content on 50 nt spanning both sides of each exon–intron junction, sequences being aligned on the consensus 5′ and 3′ splice sites. With such an alignment, information of nucleotide position according to the Open Reading Frame (ORF) of the sequences is lost in coding regions. Each junctions were studied separately within intron number classes. Large differences being observed between introns and coding regions, to further study the effect of intron presence in coding regions we removed intron sequences and concatenated CDS parts, keeping information on CDS part rank. All CDSs within an intron number class were then aligned according to their starting methionine (conserving ORF phase). Let *x_igl_* be the *l*th nt of the *g*th gene with *i* introns and coding region length equal to *L_ig_*, $l\in \{1,\ldots ,L_{ig}\}$.

Patterns of variation in *G* + *C* at nt level were studied without taking into account intron cutting up of the CDS by simply computing

(1) $$GC_{i}^{nt}(l)=\frac{\sum_{g,l\leq L_{ig}}1_{GC}(x_{igl})}{\sum_{g,l\leq L_{ig}}1},$$

where $1_{GC}(x_{igl})=1$ if $x_{igl}\in \{G,C\}$ and $1_{GC}(x_{igl})=0$ otherwise.

Intron cutting up was took into account by computing GC-content per nt position according to the CDS part the nt belongs to

(2) $$GC_{ij}^{nt}(l)=\frac{\sum_{g,l\leq L_{ig}}1_{cds_{j}}(x_{igl})1_{GC}(x_{igl})}{\sum_{g,l\leq L_{ig}}1_{cds_{j}}(x_{igl})},$$

where $1_{cds_{j}}(x_{igl})=1$ if *x_igl_* belongs to the *j*th CDS part and $1_{cds_{j}}(x_{igl})=0$ otherwise.

*GC*1, *GC*2, and *GC*3 patterns of changes at nt level are provided by subsetting over the respective codon position in the former distribution ($GC1_{ij}(3c-2)=GC_{ij}^{nt}(3c-2),GC2_{ij}(3c-1)$, and $GC3_{ij}(3c)$, with $c\in \{1,\ldots ,L_{ig}/3\}$). Codon GC-content is simply the mean of the three positions within a codon and was displayed at the second codon position leading to

(3) $$GC_{ij}^{cod}(3c-1)=$$

(3) $$\frac{GC_{ij}^{nt}(3c-2)+GC_{ij}^{nt}(3c-1)+GC1_{ij}^{nt}(3c)}{3}.$$

When a codon was interrupted by an intron, it was discarded from the computations. In all cases, GC-content was computed only when a nucleotide position was represented by at least 50 different genes.

At element level (CDS part or intron), GC-content of the different CDS parts or introns composing the genes was computed as the count of *G* and *C* nts over the total number of nts of the element and denoted $GC_{igj}^{elt}$ where *elt* indicates the type of the element (*cds* for CDS part, *I* for intron), *j* indicating the rank of the element in the gene decomposition (see fig. 1). In addition, we computed the CDS part GC-content per codon position (denoted $GC1_{igj}$ for the first, $GC2_{igj}$ for the second, and $GC3_{igj}$ for the third position within codons) as the count of *G* and *C* nts at the given codon position over the count of nts at this codon position within the element. Average CDS part (or intron) GC-content was computed by averaging over all genes within an intron number class according to CDS part (or intron) rank, as shown in the following equation for CDS part

(4) $$GC_{ij}^{cds}=\frac{1}{N_{i}}\sum_{g=1}^{g=N_{i}}GC_{igj}^{cds}.$$

#### Codon and Amino Acid Analyses

For each of the two species, we also computed CDS part codon and amino acid frequencies within classes. Stop codons as well as codons or amino acids overlapping between CDS parts were discarded. Let *nc_igj_* be the vector of the counts of the 61 codons within the *g*th CDS of the *j*th CDS part of the genes with *i* introns, codon frequencies were computed as

(5) $$COD_{ij}=\frac{\sum_{g=1}^{N_{i}}nc_{igj}}{\sum_{codons}\sum_{g=1}^{N_{i}}nc_{igj}},$$

where *COD_ij_* referred to the vector of frequencies of the 61 nonstop codons observed at the *j*th rank in genes with *i* introns. Let *naa_igj_* be the vector of the counts of the 20 amino acids (aa) within the *g*th CDS of the *j*th CDS part of the genes with *i* introns, amino acid frequencies were computed as

(6) $$AA_{ij}=\frac{\sum_{g=1}^{N_{i}}naa_{igj}}{\sum_{aa}\sum_{g=1}^{N_{i}}naa_{igj}},$$

where *AA_ij_* referred to the vector of the 20 amino acids observed at the *j*th rank in genes with *i* introns.

### Intraspecies Comparisons between Genes with or without Intron Inserted within UTR

In each species, we compared the reference data set composed of genes with no intron inserted in UTRs with each of the two data sets composed of genes having an intron inserted in their either 5′-UTR or 3′-UTR. Comparisons between the data sets were made between genes having the same number of introns inserted within their coding regions, the two data sets differing by the presence/absence of a single intron located either within the 5′- or 3′-UTRs. Within the different intron number classes, we used Welsh two-sample student tests on first (respectively, last) CDS part to test for the existence of significant differences in GC-content. In addition, to investigate for an intron specific effect, we took both the 5′-UTR data sets and the intron-free UTR data sets and sorted genes into bins according to the distance between the transcription start site and the translation start site. Within each bin, we then tested for differences in GC-content between the two sets of genes with a two-sided sign-rank test (Wilcoxon test). We made the same kind of analyses on the intron free UTR gene subsets, sorting genes into two classes (short vs. long first intron, below and above 149 and 245 nt, respectively, in *A. thaliana* and rice) and then in bins according to the distance between the transcription start site and the beginning of the second exon. In all cases, gene counts within bins were higher than 25 genes and in most cases higher than 100 genes. Finally, in the subset of paralog genes, we formed pairs of duplicated genes composed of the gene without intron inserted within UTRs and the gene with an intron inserted within either the 5′- or the 3′-UTRs. Genes from different intron number classes were pooled and two-sided Wilcoxson-paired tests were performed on CDS part and codon position GC-content. In all cases, a Bonferroni correction for multiple comparisons was also performed.

### Intraspecies Relationships between CDS Part and Whole CDS GC-Content

Whole CDS GC-content of a gene is the mean of its CDS part GC-content weighted by the proportion of the different CDS parts within the gene:

(7) $$GC_{ig}^{CDS}=\sum_{j=1}^{j=i+1}\frac{l_{ijg}}{L_{ig}}GC_{ijg}^{cds},$$

where *l_ijg_* is the length of the *j*th CDS part (j∈{1,…,i+1}) and $L_{ig}=\sum_{j=1}^{j=i+1}l_{ijg}$ is the total number of nts of the CDS of the gene of interest. By construction, a link between GC-gradient along genes and its CDS GC-content is expected.

Gradients per intron number classes were computed as

(8) $$G_{i}=\max_{1\leq j\leq i+1}(GC_{ij}^{cds})-\min_{1\leq j\leq i+1}(GC_{ij}^{cds}),$$

where $GC_{ij}^{cds}$ stand for the average CDS part GC-content of rank *j*. By construction, gradient amplitude is always positive independently of the shape of the gradient. Gradient amplitudes according to codon positions were computed in the same way.

To gauge the link between CDS part GC-content, intron number, and rank along genes, we fitted a beta regression model appropriate for the analyses of proportions using the R package betareg (Cribari-Neto and Zeileis 2010). Betareg is based on an alternative parameterization of the beta density in terms of the mean *μ* and a precision parameter ϕ estimated by maximum likelihood. It permits to perform inference with variable dispersion model by fitting in a similar fashion two submodels, one for the mean and one for the dispersion parameter. Our assumption is that for a given intron number *i* and a given rank *j* along the gene, CDS part GC-content follows a beta law $GC_{ij}^{cds}∼B(\mu_{ij},\phi_{ij})$ of mean $0<\mu_{ij}<1$ and precision $\phi_{ij}>0$. The beta regression model was fitted using the model (denoted model M1)

(9) $$GC_{ijg}^{cds}∼\mu +f_{ij}+\epsilon_{ijg}$$

treating the combination of intron number *i* and rank *j* as a factor *f_ij_* (0≤i≤14 and 1≤j≤i+1) both for the mean and the precision submodels.

To gauge the links between intron number and CDS GC-content, we made the assumption that for a given intron number *i*, CDS GC-content follows a beta law of mean $0<\mu_{i}<1$ and $\phi_{i}>0,\;GC_{i}^{CDS}∼B(\mu_{i},\phi_{i})$ and fitted a regression using the model (denoted model M2)

(10) $$GC_{ig}^{CDS}∼\mu +f_{i}+\epsilon_{ig}$$

treating gene intron number as a factor *f_i_* (0≤i≤14) both for the mean and the precision submodels.

In both cases, the link function was the logit function for the mean and the log function for the precision submodels.

In addition, we investigated the patterns of variation in variance in relationship with intron number. CDS GC-content variances were decomposed to investigate the importance of CDS part variances, covariances among CDS parts and covariances within CDS parts among codon positions. The nonsynonymous nts (denoted *F*) cannot shift either from A or T to G or C or the converse without altering the amino acid sequence of the protein and the synonymous ones (denoted *V*) are those nts for which AT↔GC shift is possible without altering the amino acid sequence. If *V_ij_* and *F_ij_* represent the respective contributions of CDS part synonymous and nonsynonymous nts to CDS part *j* GC-content of genes with *i* introns, then CDS part GC-content variance for genes with *i* introns can be written as

$$\sigma^{2}(GC_{i}^{CDS})=\underset{var.intra}{\underset{︸}{\sum_{j=1}^{j=i+1}\sigma^{2}(V_{ij})+\sum_{j=1}^{j=i+1}\sigma^{2}(F_{ij})}}$$

$$+\underset{cov.inter}{\underset{︸}{2\sum_{j=1}^{j=i}\sum_{k=j+1}^{k=i+1}\sigma (V_{ij},V_{ik})+2\sum_{j=1}^{j=i}\sum_{k=j+1}^{k=i+1}\sigma (F_{ij},F_{ik})}}$$

(11) $$+\underset{cov.VF}{\underset{︸}{2\sum_{j=1}^{j=i+1}\sum_{k=1}^{k=i+1}\sigma (V_{ij},F_{ik})}}$$

with $V_{ijg}=\frac{l_{igj}^{V}}{L_{ig}}GC_{igj}^{V}$ and $F_{igj}=\frac{l_{igj}^{F}}{L_{ig}}GC_{igj}^{F}$, where $GC_{igj}^{V}$ and $GC_{igj}^{F}$ represent the GC-contents at synonymous and nonsynonymous sites of CDS part *j* of the *g*th gene in the *i*th intron number classes, and $l_{igj}^{V}$ and $l_{igj}^{F}$ the number of each types of sites ($L_{ig}=\sum_{j=1}^{j=i+1}l_{igj}^{V}+l_{igj}^{F}$). Notice that a negative covariance between *V* and *F* decreases the overall GC-content variance.

## Results

Genomic data from two contrasted plant species, the GC-rich monocot *O. sativa* and the GC-poor eudicot *A. thaliana*, were used for a comparative study of the effect of intron number on gene GC-content. Genes with no intron located in UTRs represent more than 75% of the genes in both species and were used to describe patterns of variation linked with intron presence within CDS. We also studied two other gene subsets, composed of genes with any number of introns within CDS and a single additional intron present in either the 5′- or the 3′-UTR. These subsets were used to investigate the consequences of intron presence outside coding regions on GC-content of both introns and coding regions. We focused our analyses on exon coding parts (see Materials and Methods) and to avoid terminology confusion we discarded the term “exons” and used the term “CDS parts” for coding regions.

### CDS GC-Content Varies with Intron Number in Both Species

**Fig. 2.—:**
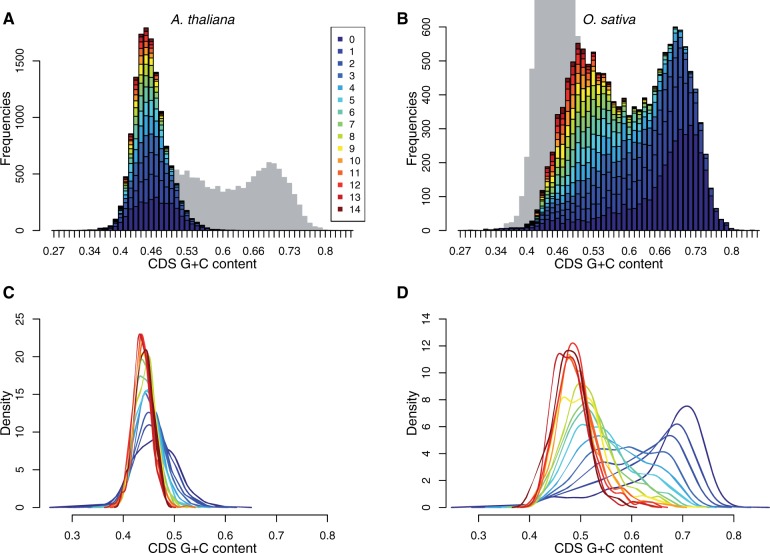
CDS GC-content distribution according to intron number in *A. thaliana* (*A*, *C*) and rice (*B*, *D*). (*A*, *B*) Gene counts according to GC-content. The contribution of intron number classes to each of the bars is indicated by the proportion of the bar of the relevant color (legend in panel *A*). In both panels, the outline of the distribution for the other species is shown in gray. (*A*) *Arabidopsis thaliana* distribution is unimodal and GC-poor. (*B*) *Oryza sativa* coding regions are globally richer in GC-content as compared with *A. thaliana* and present a bimodal distribution. In both species, GC-rich bars are mainly composed of genes with a low intron number, whereas genes with a high intron number are mainly concentrated in the GC-poor classes. (*C*, *D*) Density outlines for each intron number classes (color legend in panel *A*). In both species, GC-content varies with intron number, in location and in dispersion. The enrichment in GC-content observed in rice affects all intron number classes but is much higher in low intron number genes than in genes with high intron numbers. A large part of the bimodality in GC-content of this species appears to be linked with variation with intron number.

*Arabidopsis thaliana* genome is GC-poor, exhibiting a unimodal and homogeneous distribution in coding region GC-content (mode at 45% and standard deviation [SD]=3.2%; fig. 2*A* and *C*). The rice genome is GC-rich and highly heterogeneous displaying a bimodal distribution of CDS GC-content with two modes at 49% and 69% (SD=9.4%; fig. 2*B* and *D*). Despite large differences in GC-content between the two studied genomes, the same trends were observed at CDS scale: 1) CDS GC-content decreases toward a lower limit in GC-content with the increase in intron number and 2) the decrease in CDS GC-content is associated with a decrease in CDS GC-content variance within intron number classes. In rice, changes in GC-content with intron number are so large that they account for a large part of the bimodality observed. Because variations in GC-content according to intron number were too large to be neglected even in *A. thaliana*, genes were sorted in classes according to intron number and patterns of GC-content variation were studied within classes in all subsequent analyses.

### Introns Are Associated with Step-Gradients in Coding Region GC-Content at Nucleotide Level

**Fig. 3.—:**
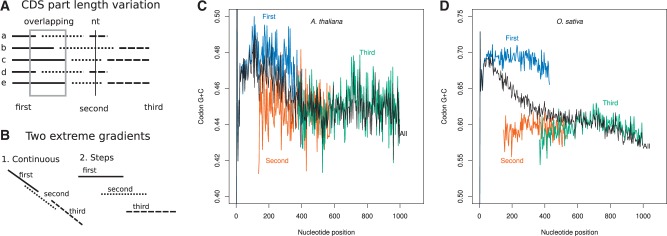
Nucleotide GC-gradient along genes with two introns. (*A*) Variations in CDS part length among genes lead to large overlap between CDS parts with different ranks. At a given nt position, GC-content is a mixture of CDS part with different ranks when intron locations are neglected. (*B*) If nt GC-content is purely due to nt position along the genes, GC-content between contiguous CDS parts should be continuous even when nt GC-content is computed by subsetting on CDS part rank. In contrast, if intron presence is associated with changes in GC-content between contiguous CDS parts, a step-gradient should arise. Notice that in both cases, a continuous gradient is expected when gene architecture is neglected. However, although in the first case both gradients will be confounded, in the second, clear differences should arise in regions of overlap. (*C*, *D*) nt GC gradients along genes with two introns when CDS parts are individualized (first CDS part: blue, second: orange, third: green) and without taking into account CDS part rank (all: black) in genes with two introns. (*C*) *Arabidopsis thaliana*. (*D*) *Oryza sativa*. In both species, differences between CDS parts are larger than oscillations within CDS parts and GC-content computed without taking into account CDS part rank is different from CDS part GC-content in regions of overlap. For clarity, only nt positions represented by at least 400 different gene sequences where plotted.

**Fig. 4.—:**
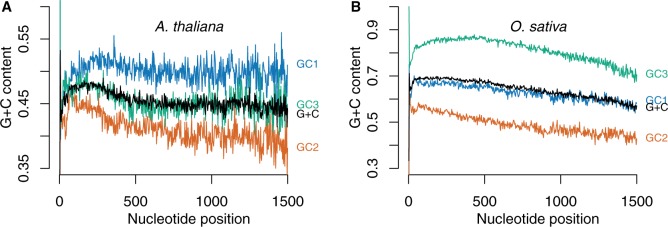
GC-content gradients in intronless genes. GC-content for each nt position from the starting methionine is indicated in blue (*GC*1), orange (*GC*2) and green (*GC*3), the resulting codon GC-content (average of the three codon positions) being plotted in black (*G* + *C*) and displayed at the second position. In both species, large differences are observed among codon positions. However compared with intronic genes (e.g., figs. 3 and 5), gradients are small. (*A*) *Arabidopsis thaliana*. (*B*) *Oryza sativa*.

**Fig. 5.—:**
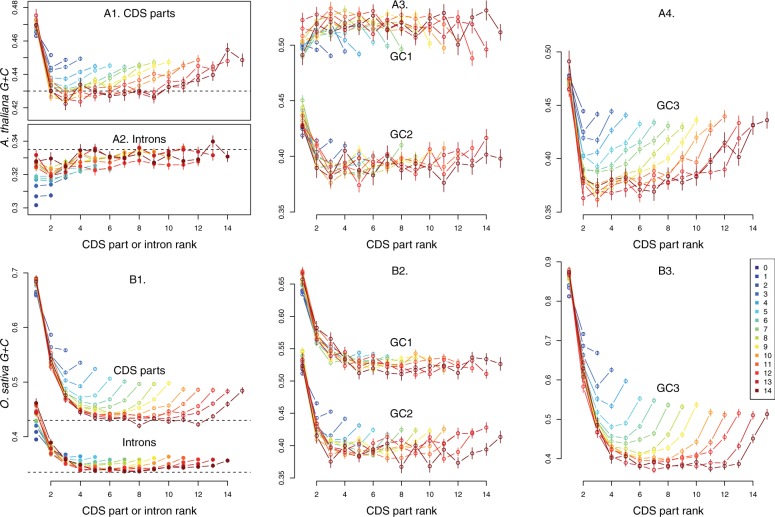
CDS part and intron GC-gradients according to rank along genes within intron number classes. In all panels, gene intron number is indicated by the colors (legend shown in panel *B*3). Bars on dots represent standard error of means. *Arabidopsis thaliana*: *A*1. Average CDS part GC-gradient. *A*2. Average Intron GC gradients. *A*3. Average CDS part *GC*1 (upper groups of lines) and *GC*2 (lower group of lines) gradients. *A*4. Average CDS part *GC*3 gradients. *Oryza sativa*: *B*1. Average CDS part (upper group of lines) and intron (lower group of lines) GC-gradients. *B*2. Average CDS part *GC*1 (upper groups of lines) and *GC*2 (lower group of lines) gradients. *B*3. Average CDS part *GC*3 gradients. The upper dashed line in the *B*1 panel is placed at the same level as the dashed line in panel *A*1. Likewise the lower dashed line in the panel *B*1 is placed at the same level as the one in the panel *A*2 indicating that both species internal introns tend to reach similar GC-content as intron number increases.

We first investigated changes in GC-content at junctions between introns and coding regions and observed sharp transitions usually larger than 10% between introns and coding regions, introns being GC-poor compared with coding regions (observed for all intronic genes; see supplementary figs. S1 and S2, Supplementary Material online). Hence, regarding nt GC-content, introns appear as holes or valleys as compared with coding regions and one can wonder whether CDS is homogeneous regardless of intron presence or whether intron presence is associated with changes in nucleotide composition. In the first case, gradients of GC-content should be continuous whereas in the second case, differences among CDS parts are expected (fig. 3*A* and *B*). To answer this question, we removed intron sequences, concatenated CDS parts of each genes keeping track of CDS part ranks, and computed GC-content per nt position according to distance from the translation start of the gene. GC-content per nt positions along the genes was compared either by summing over all the genes independently of CDS part rank or by subsetting over nt positions according to CDS part rank. Discrete changes in GC-content can be observed at least between the first and the second CDS part (fig. 3*C* and *D* for genes with two introns). The continuous gradients observed when neglecting gene architecture (black curves in fig. 3*C* and *D*) that were previously reported in several studies (Wong et al. 2002; Tatarinova et al. 2010) are artifacts caused by the transition of a number of sequences from a given CDS part rank to the next and not by a progressive change in GC-content according to codon position. As a result, in both species, 5′–3′ GC-content gradients are tightly associated with introns, suggesting that introns and CDS parts are the relevant scale of observation to study and compare GC-content gradients at gene scale. In contrast as shown in figure 4, GC-content gradients in intronless genes of both species are weak as compared with the gradients observed in intronic genes further suggesting that introns might be involved in gradients. We also investigated patterns of variation according to codon position in coding regions. In both species, large differences in GC-content among codon positions are observed. In intronic genes of both species, step-gradients are observed for all codon positions at least in 5′ gene regions (data not shown; see fig. 5 for patterns at CDS part levels).

### GC-Content Gradients Are Affected by Intron Number and Partly Explain the Genome-Wide Variation in Complete CDS GC-Content

To compare gradients along genes for different intron numbers within species, we computed GC-content for each CDS part along the genes and averaged over rank along genes within intron number classes. Structured GC-content gradients modulated by intron number are observed in both species (fig. 5). They vary in shapes and amplitudes according to the species and to the codon positions.

In both species, all CDS part gradients are U-shaped and asymmetrical except the *GC*1 gradients in *A. thaliana* that tend to be bell-shaped. Gradients arise progressively as intron number increases, appearing truncated in genes with less than two or three introns. The highest and lowest GC-content levels are observed for *GC*3 gradients in both species, average *GC*3 of first CDS part reaching up to 48% in *Arabidopsis* and 85% in GC-content in rice, respectively, and decreasing within a few steps down to 37% in both species for genes with high intron number. In both species, the gradients increase again toward the 3′-end of the CDS and stabilize at intermediate *G* + *C* levels around 44–45% in *A. thaliana* and 55% in rice in last CDS parts. Compared with *GC*3 gradients (fig. 5*A*4–*B*3), *GC*1 and *GC*2 gradients are shrunk, noisy, and less distinct whereas the resulting GC-content gradients are distinct and regular albeit less deep than *GC*3 gradients.

Finally, both the direction and the amplitude of the gradients are correlated with the direction and the importance of the changes observed in GC-content at complete CDS scale in both species (supplementary tables S3 and S4 and figs. S3–S5, Supplementary Material online, for additional information). Indeed, beta regressions of complete CDS against gene intron number and beta regressions within intron classes of CDS part GC-content rank along gene indicate that in both cases, gene structure explains a large part of the variance between genes or CDS parts in rice and a significant although modest part in *A. thaliana* (table 1).

**Table 1:** Beta Regression Fitting

Model	Df M(N)	LogLik M(N)	Chisq	*P* value	Pseudo-*R*^2^
Os: M1	240 (121)	69,186 (51,223)	35,925	$<10^{-6}$	0.52
Os: M2	30 (16)	17,748 (13,998)	7,500	$<10^{-6}$	0.38
At: M1	240 (121)	104,044 (101,653)	4,781	$<10^{-6}$	0.09
At: M2	30 (16)	27,917 (27,360)	1,114	$<10^{-6}$	0.06

Note.—Two models were fitted in *Arabidopsis thaliana* (At) and *Oryza sativa* (Os): An M1 model for the regression of CDS part GC-content as a function of rank along gene within intron number classes, and an M2 model for the regression of CDS GC-content as a function of intron number. df, degree of freedom; LogLik, LogLikelihood; Chisq, likelihood ratio Chi-squared statistic. First numbers are corresponding values for fitted models (M), numbers between brackets for NULL models (N). Pseudo-*R*^2^ provides an estimation of the goodness of fit of the model (squared correlation of linear predictor and link transformed response).

Introns are GC-poor compared with CDS parts (fig. 5*A*2–*B*1) and present gradients in the two studied species. In *A. thaliana*, for low intron number classes, average intron GC-content is mainly determined by intron number, first increasing with intron number for gene classes with few introns before stabilizing at GC-content around 32–33% for gene classes with more than five introns. In rice, U-shaped gradients modulated by intron number are observed. Like for CDS part gradients, they are truncated for low intron number and otherwise highly regular albeit the amplitudes of the gradients are small compared with rice CDS part gradients. In this species, first intron GC-contents show a distinct increase with intron number whereas last introns stabilize at 36% of *G* + *C*, GC-content in the lower part of the U stabilizing above 33%.

### Intron Presence in 5′- or 3′-UTR Modifies GC-Content Gradients

**Fig. 6.—:**
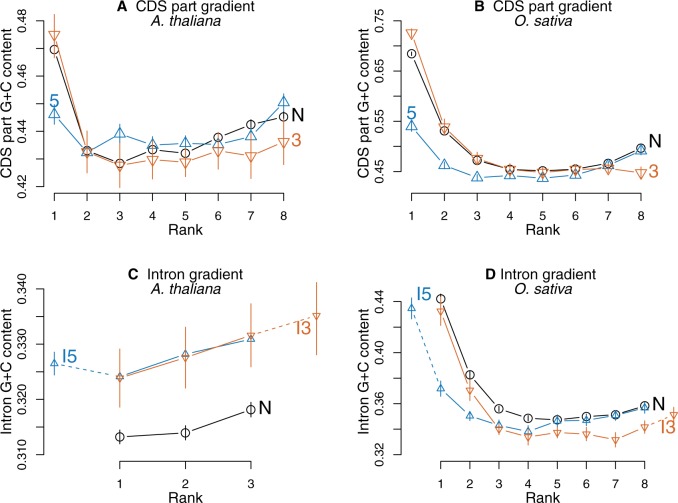
Comparison between genes having an additional intron present within one UTR with genes having no intron inserted in UTRs. In each case, the comparison is made between genes having the same number of introns within coding regions and differing only by the presence or the absence of an intron in their UTRs. N stands for no intron present in UTRs (black dots and curves), 5 stands for additional intron present in 5′-UTR (blue upper triangles and curves), 3 stands for additional intron present in 3′-UTR (orange lower triangles and curves). For intron gradients (panels *C* and *D*), solid lines connect intron ranks for introns located within CDS, whereas additional introns are connected by dotted lines (I5 stands for the additional introns present within the 5′-UTR, I3 for the additional introns present within the 3′-UTR). Bars on dots represent standard error of means. (*A*) Average CDS part GC-gradients in genes having seven introns inserted within CDS in *A. thaliana*. (*B*) Average CDS part GC-gradients of genes having seven introns inserted within CDS in *O. sativa*. (*C*) Average intron GC-gradients of genes having two introns inserted within CDS in *A. thaliana*. Intron presence in 5′- and 3′-UTR leads to a similar increase in GC-content. (*D*) Average intron GC-gradients of genes having seven introns inserted within CDS in *O. sativa*.

In both species, intron presence in 5′-UTR is associated with a decrease in 5′ external CDS part GC-content (fig. 6*A* and *B* for genes with seven introns, supplementary fig. S6, Supplementary Material online, for all studied intron numbers). In rice, a systematic decrease in GC-content is also observed when an intron is present in 3′-UTR whereas in *A. thaliana*, although decreases are generally observed they are not always significant. Similar patterns are observed for each codon position taken separately (supplementary figs. S7–S9 and tables S5–S12, Supplementary Material online). Notice than the other extremity of the gradient remains unaffected in most cases.

Regarding intron gradients, the additional introns are integrated into the intron gradients that look otherwise normal in both species (fig. 6*C* and *D* for genes with two introns inserted with the CDS in *A. thaliana* and genes with eight introns inserted within CDS in rice, supplementary fig. S6, Supplementary Material online, for all intron numbers). In *A. thaliana*, the presence of an additional intron either in 5′- or in 3′-UTR is associated with an overall increase in GC-content of all the introns. In rice, the UTR’s intron is added to the gradient at the relevant extremity whereas the next intron (the second when the intron is inserted in the 5′-UTR or the penultimate when the intron is located in the 3′-UTR) presents a significant decrease in GC-content that drives it at a *G* + *C* level similar to a second or a last intron of genes with no intron present in their UTRs. Again, this pattern is consistent for all intron number classes with low intron numbers.

Similar results were obtained in each species with a subset of pairs of paralog genes differing by the presence/absence of an intron in the 5′- or 3′-UTR of one member of each pair, both members of the pairs having the same number of introns inserted within coding regions. When paired Wilcoxon signed-rank tests indicated the existence of significant differences, they were in the same direction as those described above in the two species confirming the hypothesis that changes in intron structure are implicated in the changes in GC-content (see supplementary tables S13 and S14, Supplementary Material online).

These results suggest that intron presence is associated with some processes impeding the increase in GC-content observed in external gene regions to progress into the internal regions of genes.

### Introns Have a Specific Impact on nt Composition of Coding Regions

**Fig. 7.—:**
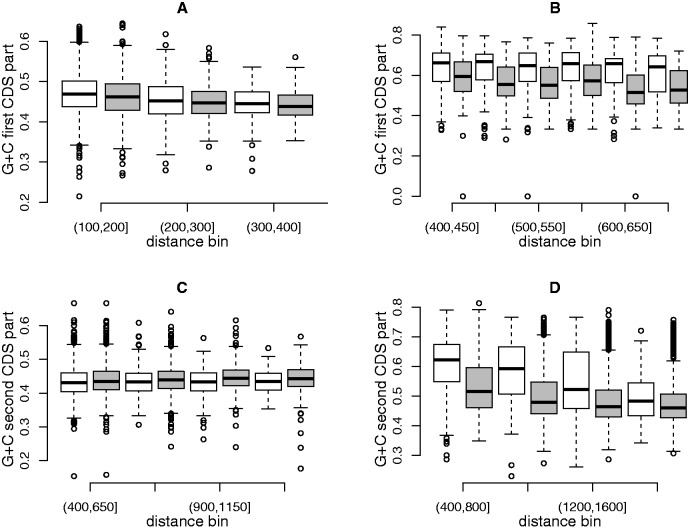
Intron-specific effect. (*A*, *B*) Comparison between genes differing by the presence/absence of an intron within the 5′-UTR. Six bins of distance between the transcription start site and the translation start sites were formed for genes with no intron in UTRs (white) and genes with a single intron in the 5′-UTR (gray). For each bins, the boxplots show the GC-content of the first CDS part and in both species a decrease is observed between UTR alone and UTR plus intron. (*A*) *Arabidopsis thaliana*: Sign-ranks tests within bins were all significant (*P* < 0.05, in most cases *P* < 0.001) except for the two last bins. (*B*) *Oryza sativa*: Sign-ranks tests within bins were all significant (*P* < 0.0001 except for the last bin *P* < 0.01). (*C*, *D*) Intron length threshold effect. Genes with no intron inserted within UTR were sorted into two groups according to the length of their first intron (below 150 nt: white; above 150 nt: gray) for *A. thaliana* and (below 250 nt: white; above 250 nt: gray) for *O. sativa*. For each bins, the boxplots show the GC-content of the second CDS part. (*C*) *Arabidopsis thaliana*: An increase is observed in genes with long introns, sign-ranks tests within bins being all significant (*P* < 0.005). (*B*) *Oryza sativa*: A decrease is observed in genes with long introns, sign-ranks tests within bins being all significant (*P* < 0.0001 except for the last bin *P* < 0.05).

To test whether the changes described above are purely due to the addition of a given number of nts that takes away coding regions from transcription start sites (TSS) or whether there is a specific intron effect, we performed two different kinds of comparisons. First, we compared first CDS part GC-content between genes having no intron inserted within their 5′-UTRs and genes having an intron inserted within their 5′-UTRs and similar distances between the TSS and the start codon. We sorted each group of genes into six bins according to the distance between first CDS part and TSS (when no intron is inserted within 5′-UTR this distance is equal to the 5′-UTR length whereas when an intron is inserted, it is equal to the sum of the two 5′-UTR part lengths plus the length of the intron) and looked for a difference between the two groups of genes within size bins. Indeed, a significant decrease in first CDS part GC-content is observed in both species when an intron is present within the 5′-UTR compared with pure 5′-UTR of comparable length suggesting that introns have a larger impact than 5′-UTR sequences (fig. 7*A* and *B*). Second, in the subsample of genes with no intron inserted within UTRs we sorted genes into two classes according to the length of their first introns (shorter or longer than 150 nt in *A. thaliana* and 250 nt in rice, see supplementary figs. S10 and S11, Supplementary Material online, for additional information) and compared GC-content of the second CDS part in genes having a similar distance between TSS and the beginning of the second CDS part (this distance is equal to the sum of the 5′-UTR, first CDS part, and first intron lengths). Again, an intron-specific effect is detected in both species (fig. 7*C* and *D*). In rice, the effect of first intron length on second CDS part GC-content takes the form of a huge decrease in second CDS part GC-content which appears to depend in a threshold way on the length of the first intron and can even affect all downstream CDS parts or introns in genes with few introns (see supplementary fig. S11, Supplementary Material online). In *A. thaliana*, a weak albeit significant increase of second CDS part GC-content is observed between short and long introns (fig. 7*C*). All these results suggest that in both species, intron presence has a specific impact on coding region GC-content that differs from other types of regions (5′-UTR or coding regions).

### GC-Content Variation Is Constrained in Internal Gene Regions

**Fig. 8.—:**
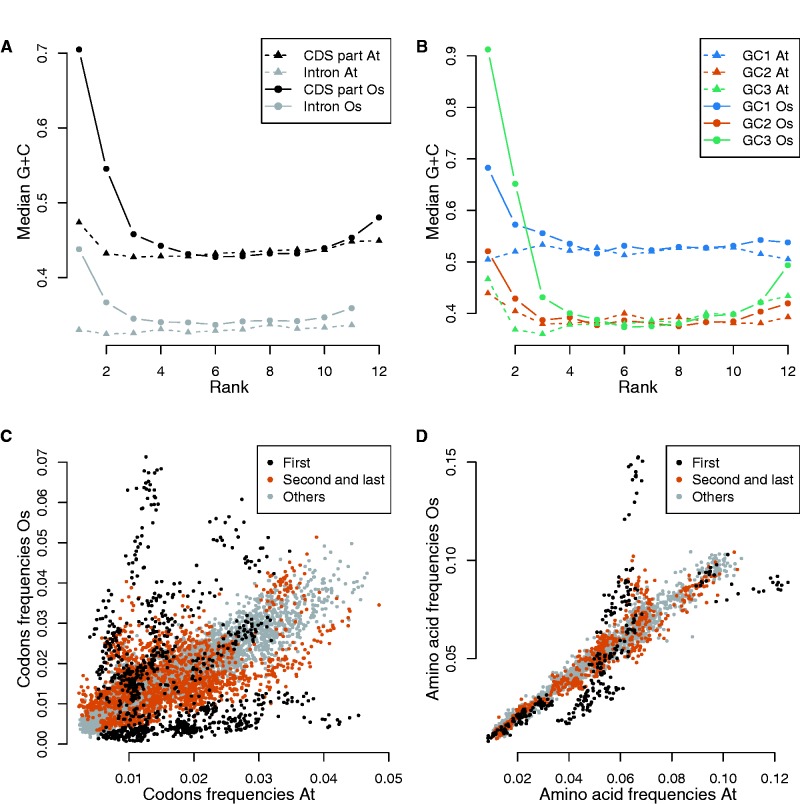
Comparisons of GC-content patterns of variation along genes within and between genomes. (*A*) Gradients in median GC-content for CDS part (black) and intron (gray) GC-content according to rank along genes in *A. thaliana* (triangle and dashed lines) and *O. sativa* (dots and plain lines) in genes with 11 introns. (*B*) *GC*1 (first position within codon, blue), *GC*2 (second position within codon, orange), *GC*3 (third position within codon, green) median gradients. All gradients differ in amplitude and in shape for each position within codons as well as for all nt positions within codons and introns. The central regions of gradients are strikingly close among rank along genes and between species, whereas external regions differ markedly especially in the 5′ regions. (*C*) Codon frequency comparisons between *A. thaliana* and *O. sativa*. Each dot represents the frequency of a codon for a given CDS part and gene intron number in *A. thaliana* on the *x*-axis and *O. sativa* on the *y*-axis. (*D*) Amino acid frequency comparisons between *A. thaliana* and *O. sativa*. Each dot represents the frequency of an amino acid for a given CDS part and gene intron number in *A. thaliana* on the *x*-axis and *O. sativa* on the *y*-axis. First CDS parts are plotted in black, second and last CDS parts in red, and all other CDS parts are plotted in gray.

Despite a strong difference in their overall GC-content, *A. thaliana* and rice exhibit common trends. Within each genome, internal introns or CDS parts in genes with high intron numbers have similar GC-content whereas external gene regions are GC-richer than internal regions (fig. 8*A* and *B*). Indeed, GC-content median gradients are all almost flat in the internal regions of genes with many introns and do not differ between the two species (fig. 8*A* for genes with 11 introns). Likewise, a similar pattern is observed for each codon positions (fig. 8*B*). In contrast, large differences among external CDS parts or introns and among species are observed. Hence, introns appear to delimit gene space into three regions, an internal region characterized by a conservation of GC-content levels among genes within a genome but also between species and two external regions submitted to other, species-specific, factors. As all codon positions form gradients along the genes, one can expect to observe also variation in codon and amino acid frequencies linked with intron number and rank along genes. Indeed, there are few differences between *A. thaliana* and rice in internal regions of genes for both codon and amino acid frequencies whereas a strong difference for both is evidenced at both ends of the genes (fig. 8*C* and *D*). Codon and amino acid variations in frequencies are consistent with GC-content patterns of variation and vary according to CDS part rank along the gene in a genome-dependent manner in external gene regions, whereas internal gene regions tend to be similar even between species.

**Fig. 9.—:**
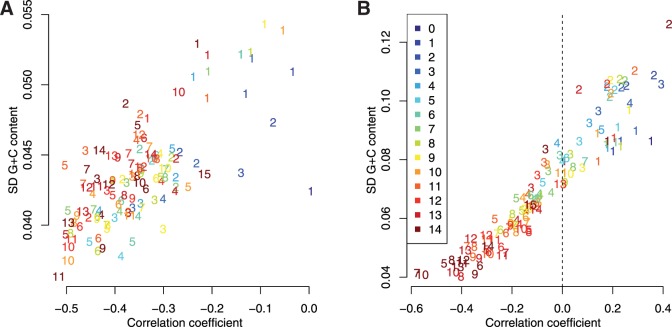
GC-content SD within CDS parts versus correlation among nonsynonymous and synonymous sites within intron number classes. Points were replaced by number indicating the CDS part rank, the colors indicating the gene intron number (legend in panel *B*). In both species, as intron number increases correlations coefficients and SD are decreasing for a given rank. The decreases in both correlation coefficient and SD are more important in internal CDS parts. (*A*) *Arabidopsis thaliana*, all correlation coefficients are negative. (*B*) *Oryza sativa*, correlation coefficients vary according to intron number and rank from positive (in genes with low intron numbers or in external CDS parts of genes with high intron numbers) to negative (in central regions of genes with high intron numbers).

To further study the different properties of external and internal regions, we analyzed the variance in GC-content and correlations between CDS part ranks along genes. We observed a lower variance in GC-content for internal CDS parts (fig. 9) and that the variance of the CDS part GC-content is lower than expected by the variances of the three codons positions assumed to be independent (see supplementary fig. S12, Supplementary Material online), suggesting constraint on GC-content. To study correlations within CDS parts, we computed Pearson correlation coefficients between GC-content at synonymous and nonsynonymous sites (see precise definition in Materials and Methods). In *A. thaliana*, the correlations are negligible in genes with low intron number but become more and more negatives in internal CDS parts as gene intron number increases (fig. 9*A*). In rice, they vary from largely positive in first and second CDS parts to largely negative in internal regions of genes with high intron numbers (fig. 9*B*). The decomposition of CDS variance indicates that these changes in sign and value of correlations along genes contribute to roughly a one-third decrease in CDS GC-content variances between genes with low and genes with high intron number in both species (see supplementary tables S15 and S16, Supplementary Material online). Although positive correlations between positions were already reported (e.g., Serres-Giardi et al. 2012), properly taking intron structure into account revealed a more complex pattern along the genes with negative correlations in internal CDS parts, which were not previously documented, as far as we know.

## Discussion

In the two studied species, both intron number and location appear as strong structuring factors for GC-content. Within genes, GC-content in coding regions is organized into step gradients delineated by introns and modulated by intron number. Intron GC-content also forms gradients varying with intron number and intron rank along the genes. For a given type of gene region, variation in intron number is mainly associated with variation in gradient amplitude, leading to the formation of highly regular patterns of variation along the genes. In both rice and *A. thaliana*, a mechanistic consequence of CDS part organization in U-shaped gradients modulated according to intron number is the existence of a negative correlation between gene intron number and complete CDS GC-content, the strength of the correlation depending positively on the steepness of the gradients. Gene intron number is then expected to be a strong determinant of CDS GC-content in GC-rich genomes and less important in GC-poor genomes. Indeed, the Spearman correlation coefficient between CDS GC-content and gene intron number is equal to −0.62 in rice whereas in *A. thaliana* it is equal to −0.25 in agreement with the results shown in table 1.

Both gene intron number and the precise gene intron–exon architecture are required to properly describe patterns of variation in GC-content along the genes. Indeed, neglecting gene intron number or intron structure leads at best to underestimate GC-content patterns of variation and can even be misleading. For example in *A. thaliana*, no gradient along genes was detected when pooling all genes (Wong et al. 2002) whereas genes with several introns show clear *GC*3 gradients and up to 15% differences in GC-content between outer and inner CDS parts. Likewise, neglecting intron location leads to continuous gradients of smaller amplitudes and hides the sharp discontinuity between CDS parts that depends on CDS rank. Another example is the correlation between synonymous and nonsynonymous nts in rice. They are positives at CDS scale in all intron number classes whereas they vary from largely positives or null in external CDS parts to largely negatives in internal CDS parts of genes with high intron number. Those three examples demonstrate that at least for GC-content, neglecting intron number can lead to fallacious interpretations. As GC-content is known to be correlated with many genomic features such as recombination rates (Choi et al. 2013; Hellsten et al. 2013), methylation patterns (Chodavarapu et al. 2010; Takuno and Gaut 2013), splicing mechanisms (Amit et al. 2012), and nucleosome positioning (Tillo and Hughes 2009), one can wonder to which extent those genomic features also correlate with intron number and rank along genes.

A range of observations suggest that our results might extend to most angiosperms. The differences between coding region and intron GC-content are described in several eudicot and monocot species (Goodall and Filipowicz 1991; Carels and Bernardi 2000). *GC*3 CDS part gradients similar to those observed in rice and *A. thaliana* have been described in four other angiosperm species, two grasses, *Zea mays* and *Sorghum bicolor* and two rosids, *Glycine max* and *Populus trichocarpa* (Qin et al. 2013, in this article the gradients shown are in (1−GC3)/GC3). Furthermore, the association between GC-content richness, increase in heterogeneity between genes, and steepness of GC-content gradients is conserved at angiosperm scale (Serres-Giardi et al. 2012). This association which arises naturally as consequence of U-shaped gradients strongly suggests that the processes occurring in two widely divergent species such as rice and *A. thaliana* might also take place in other plant species, although further studies are required to confirm the generality of our findings.

Patterns of variation in GC-content along genes are highly complex in both species. Generation and maintenance of such complexity require a diversity of evolutionary pressures (combination of mutation, selection, and drift). Indeed, patterns of variation in GC-content along genes within each genome as well as between the two studied genomes suggest that opposite forces are shaping GC-content at gene scale. In gene external coding regions, not only GC-content but also correlations between synonymous and nonsynonymous nts tend to increase, the strength of this effect depending on the species. In internal gene regions, GC-content is low in both species, leading to a large overlap of GC-content distributions in internal regions of intronic genes of the two species. In addition, correlations between synonymous and nonsynonymous nucleotides in coding regions of intronic genes of both species become largely negatives. Neither drift nor mutation is expected to produce negative correlations among codon positions and gBGC is expected to produce positive correlations because it affects all positions in the same direction. To our knowledge, stabilizing selection on GC-content appears as the only phenomenon able to produce these negative correlations within internal CDS parts.

The cause of this potential stabilizing selection is unknown but it could be related to chromatin structure and/or splicing processes. In eukaryotes including *A. thaliana* (Andersson et al. 2009; Choi et al. 2013), nucleosome occupancy is higher in coding regions and tightly linked with GC-content (Schwartz et al. 2009; Tilgner et al. 2009; Tillo and Hughes 2009), and in yeast, nucleosome occupancy has been suspected to lead to stabilizing selection in GC-content (Kenigsberg et al. 2010). Likewise, differences in GC-content between introns and CDS parts are described as promoting intron recognition and splicing in *A. thaliana* (Goodall and Filipowicz 1991; Amit et al. 2012; Gelfman et al. 2013), in maize (Carle-Urioste et al. 1997; Ko et al. 1998; Clancy and Hannah 2002) but also in other eukaryotes (Amit et al. 2012). Both factors could thus stabilize both CDS part and intron GC-content in plants and contribute to maintain the switch-back patterns with GC-rich coding regions alternating with GC-poor introns.

As described in Glémin *et al.* (2014), gBGC driven by recombination gradient could be involved to produce the species-specific increase in GC-content in external gene regions. Indeed in two plant species (*Mimulus guttatus* and *A. thaliana*), recombination gradients similar to GC-content gradients are observed (Choi et al. 2013; Hellsten et al. 2013) and in maize, patterns of gene conversion at the bronze (*bz*) locus are polarized, the sites located within 150 bp of the start and the stop codons being converted more frequently than sites located within the middle of the genes (Dooner and He 2014). If gBGC is responsible for the changes in external CDS part GC-content, our results suggest that introns could act as a barrier to recombination or to the extension of conversion tract through the middle of genes. In agreement with this hypothesis, recombination rates were found to be lower in introns than in CDS parts in *M. guttatus* (Hellsten et al. 2013).

The statistical association between variations in GC-content and intron presence is too systematic to be accidental. An intron barrier effect hypothesis provides a simple explanation to the peculiarities of GC-content patterns of variation observed in the two studied species. Indeed, in the context of the association of 1) a process causing the increase in GC-content in external gene regions, 2) constraints on GC-content in gene central regions of intronic genes, and 3) an intron barrier effect preventing the spread of the increase in GC-content in internal gene regions, CDS part U-shaped gradients appear as consequences of intron presence, whereas intron absence or low number of introns explain why genes with no or a few introns are different from the others in gradient shapes and amplitudes. It can also explain why gradients are modulated by intron number in a highly repeatable manner and why codon positions are affected in a coordinated way, the intron barriers passively dividing genes into two domains submitted to different evolutionary regimes. Furthermore, it is compatible with the changes in gradients observed when introns are present in UTRs and the decrease in CDS GC-content variance with gene intron number. In contrast, if introns were not involved in GC-content patterns of variation, the tight link between intron presence and GC-content variation would require that both CDS parts and introns are affected by the same evolutionary pressures in different yet highly coordinated ways. In both cases, studying the link between GC-content and the intron–exon architecture of the genes in other plant species will provide valuable insights into plant gene evolution.

## Supplementary Material

Supplementary tables S1–S16 and figures S1–S10 are available at *Genome Biology and Evolution* online (http://www.gbe.oxfordjournals.org/).

Supplementary Data
