# Supplementary material for: Introns Structure Patterns of Variation in Nucleotide Composition in Arabidopsis thaliana and Rice Protein-Coding Genes
Source: Genome Biol Evol. 2015 Oct 7;7(10):2913–28. doi: 10.1093/gbe/evv189 (PMC4684703; doi:10.1093/gbe/evv189)
Supplement: Supplementary Data [file supp_7_10_2913__index.html]

Introns structure patterns of variation in nucleotide composition in Arabidopsis thaliana and rice protein-coding genes — Introns Structure Patterns of Variation in Nucleotide Composition in Arabidopsis thaliana and Rice Protein-Coding Genes — Supplementary Data 

# Introns Structure Patterns of Variation in Nucleotide Composition in *Arabidopsis thaliana* and Rice Protein-Coding Genes

## Supplementary Data

files

- Supplementary Data - pdf file
