# Supplementary material for: Introns Structure Patterns of Variation in Nucleotide Composition in Arabidopsis thaliana and Rice Protein-Coding Genes
Source: Genome Biol Evol. 2015 Oct 7;7(10):2913–28. doi: 10.1093/gbe/evv189 (PMC4684703; doi:10.1093/gbe/evv189)
Supplement: Supplementary Data [file supp_evv189_Supp_Ressayre_corr.pdf]

Supplementary File : Introns structure patterns of variation in nucleotide composition in *Arabidopsis thaliana* and rice protein-coding genes

Adrienne Ressayre, Sylvain Glémin, Pierre Montalent, Laurana Serres-Giardi, Christine Dillmann and Johann Joets

September 8, 2015

1 Intron distribution within genes in both species

Within each of the two studied genomes, only gene models having a 5' and a 3' UTR and at most a single intron inserted within either their 5' or their 3' UTR independently of the number of introns inserted within their CDS were kept. Three groups of genes were then formed, those with no introns inserted within their UTRs (Intron-Free UTR or IFU), those with an intron inserted within their 5' UTR and no intron inserted within their 3' UTR and those with no intron inserted within their 5' UTR and an intron inserted within their 3' UTR. As shown in table S1, gene counts within intron number classes are very similar in the two species for genes with more than two introns. For intronless genes or genes with a single intron, large differences are observed.

	A. thaliana			*O. sativa*		
Intron	IFU	5' UTR	3' UTR	IFU	5' UTR	3' UTR
0	3060	399	101	3878	489	296
1	1935	282	66	2299	334	159
2	1583	294	49	1647	250	102
3	1232	271	37	1173	251	72
4	1095	211	31	988	213	65
5	855	159	17	802	195	60
6	796	176	25	710	160	60
7	546	168	29	600	124	42
8	520	122	26	497	118	57
9	439	85	17	428	96	41
10	317	83	13	332	67	37
11	298	74	17	268	69	22
12	242	51	12	236	52	14
13	193	42	7	180	60	25
14	135	29	9	125	31	12
Total	13246	2446	456	14163	2509	1037

Table S1: Gene counts according to gene intron number in the six different datasets from *A. thaliana* and *O. sativa*. The IFU column indicates gene counts for genes with no introns in UTRs. The 5' UTR column indicates gene counts for genes with a single intron in the 5' UTR and no intron in the 3'. The 3' UTR column indicates gene counts for genes with no intron in the 5' UTR and a single intron inserted in the 3'UTR.

In each species, we also retrieved the orthologs set of genes available on the MSU site at `http://rice.plantbiology.msu.edu/annotation_pseudo_ortho.shtml`. This dataset contains for both species the genes that are orthologous within a genome (duplicated genes) and we used it to produce in each species an additional dataset composed of duplicated genes which differ by the presence/absence of a single additional intron inserted either in the 5' UTR or 3' UTR (Supplementary table S2).

	A. thaliana		*O. sativa*	
Intron	5' UTR	3' UTR	5' UTR	3' UTR
0	21	11	11	7
1	15	16	11	1
2	41	7	18	5
3	22	8	6	1
4	18	7	6	6
5	11	5	6	6
6	11	4	4	2
7	11	2	7	1
8	13	1	6	4
9	14	3	5	4
10	15	-	1	-
11	7	-	2	-
12	5	-	5	-
13	3	-	7	-
14	1	-	2	-
Total	208	64	97	37

Table S2: Composition of the related set of genes in *A. thaliana* and in *O. sativa*. Distributions of intron number in the coding sequences of pairs of duplicated genes differing in the presence/absence of introns in their 5' UTR or their 3' UTR.

2 Patterns of variation in $G + C$ content at element scale

2.1 Transitions between CDS parts and introns

Variation between coding regions and introns were investigated by computing $G + C$ content on 50 nucleotides spanning both sides of each exon-intron junction, sequences being aligned on the consensus 5' and 3' splice sites. With such an alignment, information of nucleotide position according to the Open Reading Frame (ORF) of the sequences are lost in coding regions. For all exon-intron junctions along genes, large differences (generally larger than 10%) among coding regions and introns $G + C$ content are observed, the transitions between the two types of sequences being always abrupt, occurring within the few nucleotides surrounding the splicing sites. Compared with the large intron/coding region differences, within each type of regions only small oscillations or small trends of variation are observed between consecutive nucleotides. In both species, patterns are similar among intron number classes to those observed in genes with six introns inserted in coding regions and no intron inserted in UTRs (fig. S1 and S2).

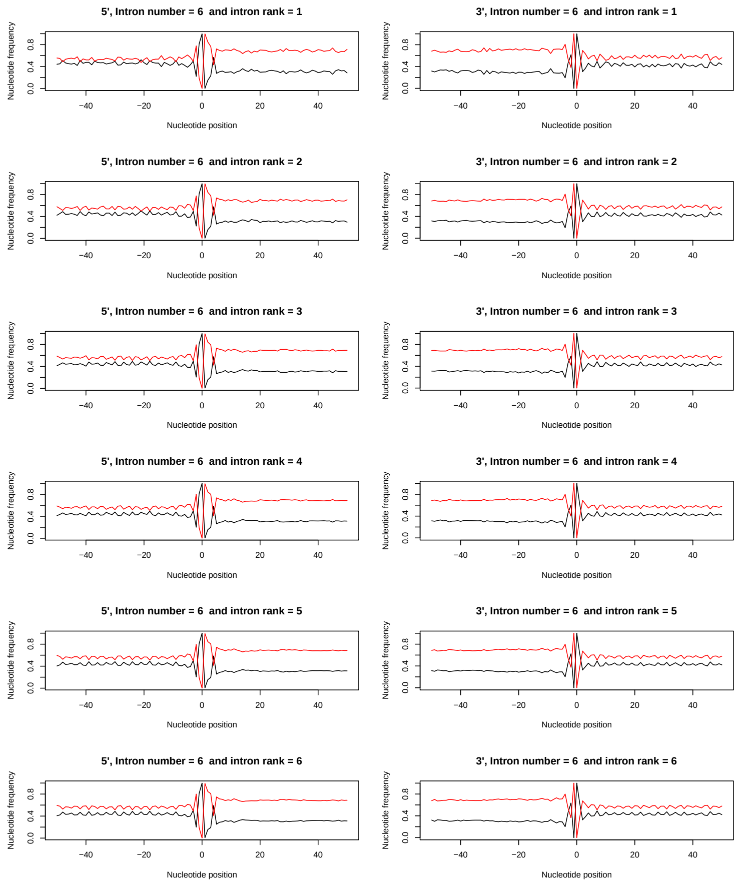

Figure S1: $G + C$ content in the 100 nucleotides surrounding the splice sites in *A. thaliana* in genes with six introns according to rank of the elements along the genes. Left column : 5' splice site (0 indicates the first nucleotide of the intron). Right column: 3' splice site (0 indicates the last nucleotide of the intron). Red: $A + T$ content, black: $G + C$ content. Introns are $G + C$-poor compared with coding regions and the transitions are sharp.

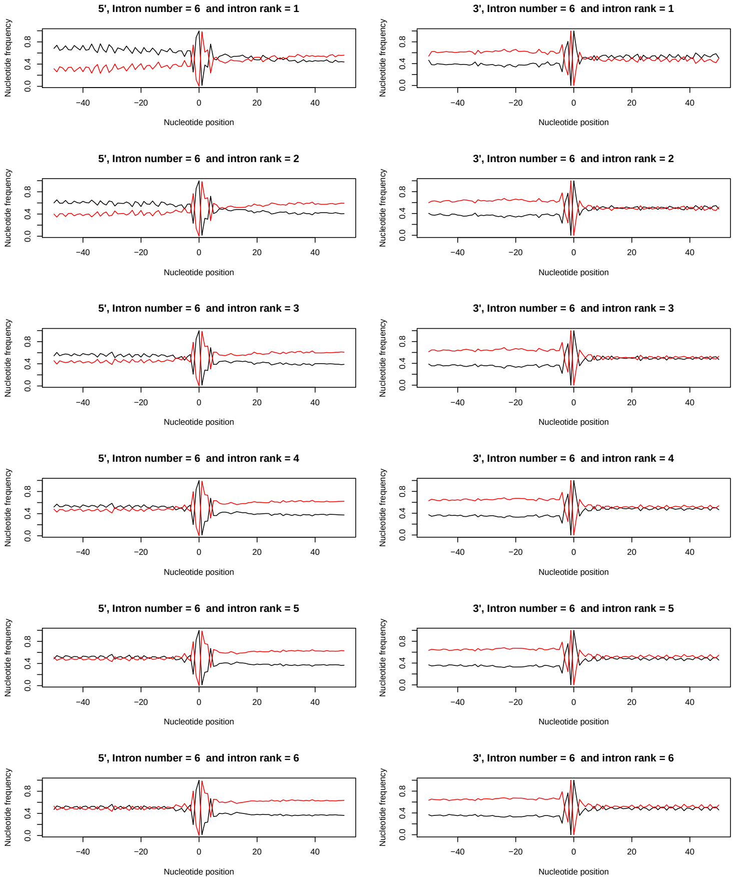

Figure S2: $G+C$ content in the 100 nucleotides surrounding the splice sites in *O. sativa* in genes with six introns according to rank of the elements along the genes. Left column : 5' splice site (0 indicates the first nucleotide of the intron). Right column: 3' splice site (0 indicates the last nucleotide of the intron). Red: $A+T$ content, black: $G+C$ content. Introns are $G+C$-poor compared with coding regions and the transitions are sharp.

2.2 CDS part gradients

Intron	$G + C$		$GC1$		$GC2$		$GC3$	
number	Median	Grad	Median	Grad	Median	Grad	Median	Grad
1	0.46	0.014	0.5	0.0049	0.41	0.0046	0.46	0.033
2	0.45	0.025	0.5	0.012	0.41	0.023	0.45	0.053
3	0.45	0.023	0.51	0.026	0.41	0.03	0.44	0.055
4	0.45	0.031	0.5	0.021	0.41	0.044	0.43	0.067
5	0.45	0.036	0.51	0.023	0.41	0.043	0.42	0.084
6	0.44	0.036	0.51	0.031	0.41	0.058	0.41	0.081
7	0.44	0.041	0.51	0.035	0.41	0.065	0.41	0.08
8	0.44	0.042	0.52	0.029	0.4	0.059	0.4	0.099
9	0.44	0.048	0.52	0.026	0.41	0.048	0.4	0.11
10	0.44	0.044	0.51	0.024	0.4	0.057	0.4	0.092
11	0.44	0.043	0.52	0.028	0.4	0.05	0.4	0.1
12	0.44	0.043	0.52	0.043	0.4	0.054	0.39	0.1
13	0.44	0.052	0.51	0.031	0.41	0.046	0.39	0.11
14	0.44	0.047	0.52	0.04	0.4	0.051	0.4	0.12

Table S3: Gradient amplitude in genes with no intron inserted within UTR in *A. thaliana*. Gene intron number is indicated in the first column, CDS $G + C$ content median and gradient amplitude per intron number classes being provided for $G + C$ content and per codon positions.

Intron	$G + C$		$GC1$		$GC2$		$GC3$	
number	Median	Grad	Medan	Grad	Median	Grad	Median	Grad
1	0.65	0.074	0.62	0.062	0.48	0.046	0.84	0.12
2	0.62	0.11	0.6	0.081	0.47	0.075	0.78	0.17
3	0.59	0.15	0.59	0.098	0.46	0.1	0.7	0.26
4	0.56	0.19	0.58	0.12	0.45	0.12	0.64	0.33
5	0.54	0.2	0.57	0.12	0.44	0.13	0.59	0.37
6	0.52	0.22	0.57	0.12	0.44	0.15	0.57	0.41
7	0.52	0.23	0.56	0.12	0.44	0.15	0.55	0.43
8	0.51	0.25	0.56	0.14	0.44	0.16	0.53	0.45
9	0.5	0.24	0.56	0.14	0.43	0.16	0.5	0.45
10	0.49	0.24	0.55	0.13	0.42	0.14	0.5	0.47
11	0.49	0.26	0.55	0.15	0.42	0.14	0.49	0.49
12	0.49	0.25	0.55	0.16	0.42	0.15	0.48	0.48
13	0.48	0.26	0.54	0.16	0.43	0.14	0.45	0.5
14	0.48	0.26	0.55	0.15	0.41	0.16	0.47	0.5

Table S4: Gradient amplitude in genes with no intron inserted within UTR in *O. sativa*. Gene intron number is indicated in the first column, CDS $G + C$ content median and gradient amplitude per intron number classes being provided for $G + C$ content and per codon positions.

3 CDS $G + C$ content according to codon position and CDS part gradients

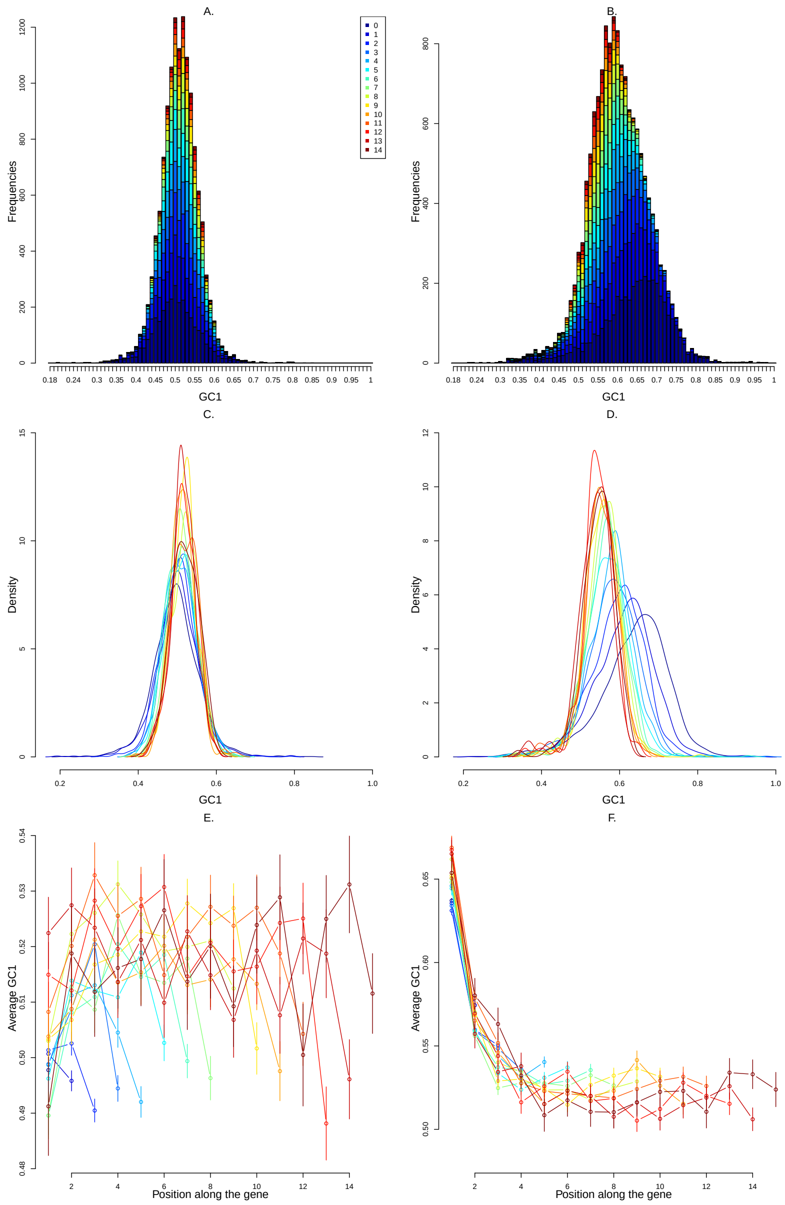

Figure S3: $G + C$ content on the first position within codons ($GC1$) patterns of variation in *A. thaliana* (left column) and *O. sativa* (right column). In all panels, gene intron number is indicated by the colors (legend in panel A). AB: Histograms of CDS $GC1$. The contribution of the intron number class to each of the bars is indicated by the proportion of the bar of the relevant color. CD: density outlines of CDS $GC1$ according to intron number. EF: Patterns of variation in CDS part average $GC1$ according to rank along genes within intron number classes (colors). Vertical bars on dots indicate standard error of means. Unlike most other gradients, $GC1$ is bell-shaped in *Arabidopsis* and as expected if gradient are important to explain CDS patterns of variation, both densities and histograms show that CDS $GC1$ increase with intron number.

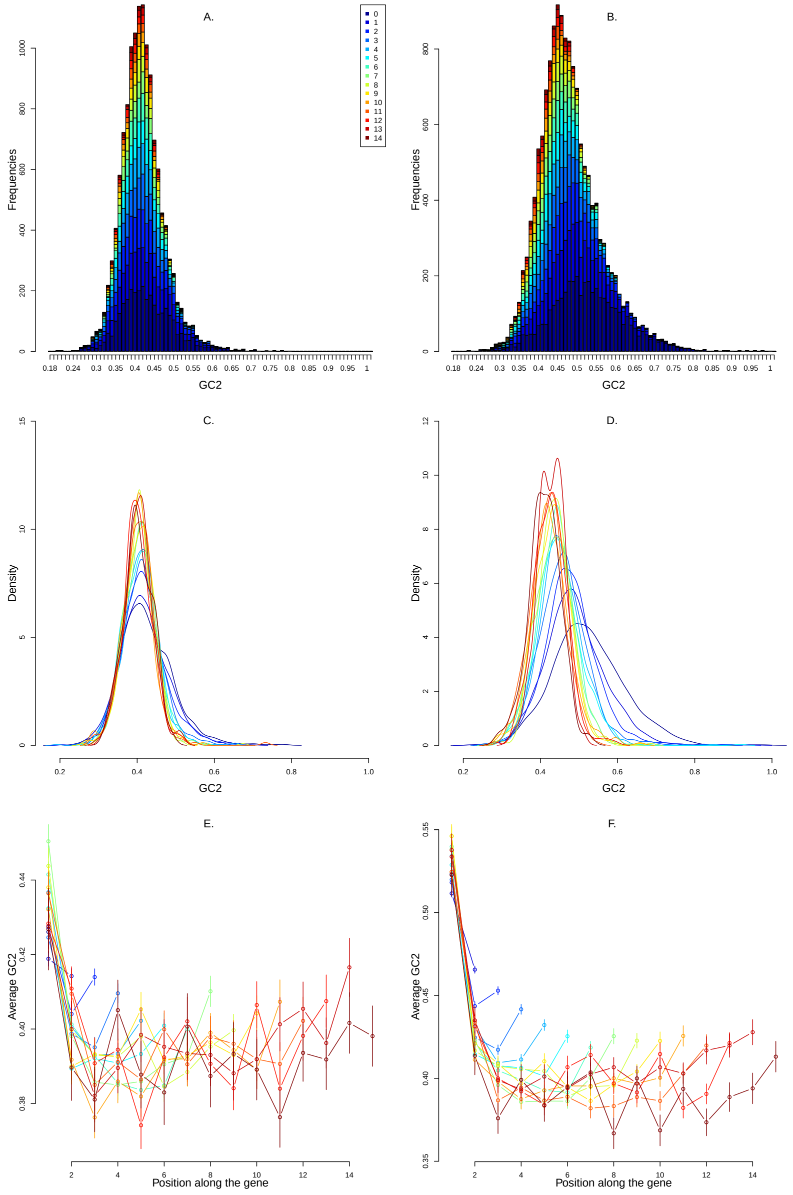

Figure S4: $G{+}C$ content on the second position within codons ($GC2$) patterns of variation in *A. thaliana* (left column) and *O. sativa* (right column). In all panels, gene intron number is indicated by the colors (legend in panel A). AB: Histograms of CDS $GC2$. The contribution of the intron number class to each of the bars is indicated by the proportion of the bar of the relevant color. CD: density outlines of CDS $GC2$ according to intron number. EF: Patterns of variation in CDS part average $GC2$ according to rank along genes within intron number classes (colors). Vertical bars on dots indicate standard error of means. Gradients are noisy and intron number classes are indistinct but U-shaped at least in low intron number classes.

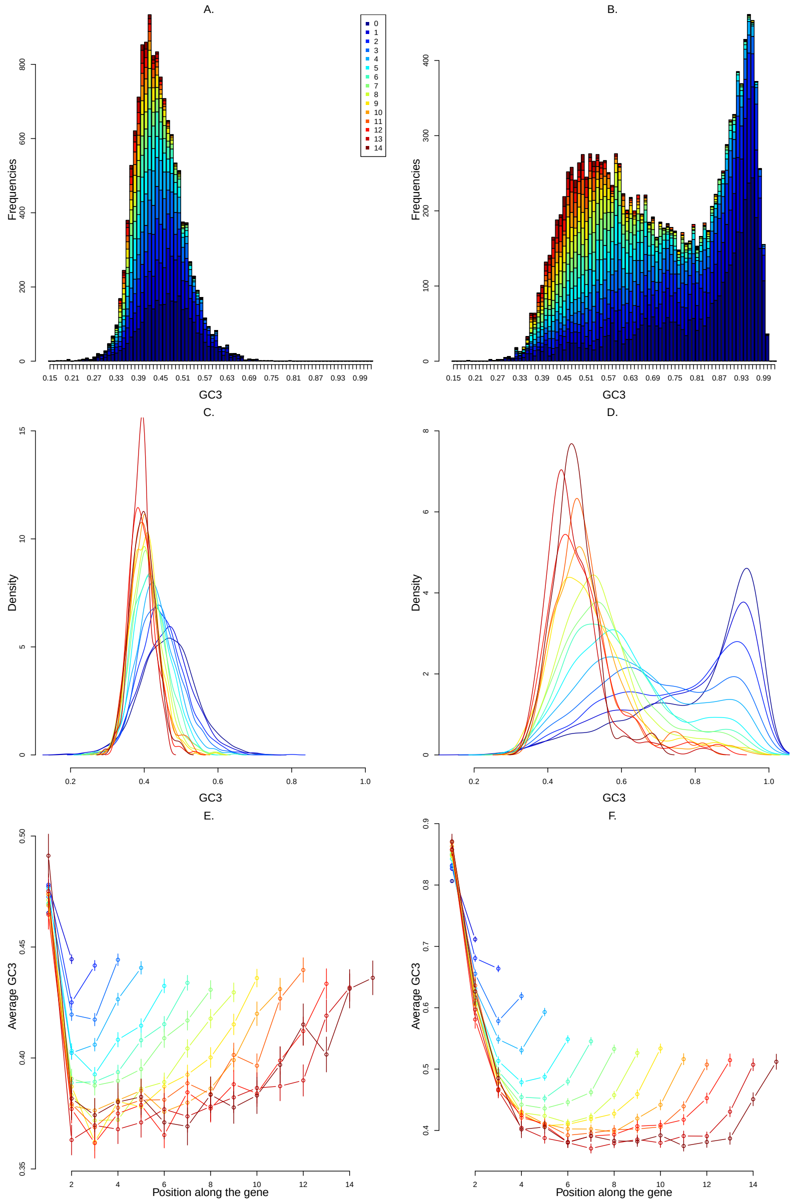

Figure S5: $G + C$ content on the third position within codons ($GC3$) patterns of variation in *A. thaliana* (left column) and *O. sativa* (right column). In all panels, gene intron number is indicated by the colors (legend in panel A). AB: Histograms of CDS $GC3$. The contribution of the intron number class to each of the bars is indicated by the proportion of the bar of the relevant color. CD: density outlines of CDS $GC3$ according to intron number. EF: Patterns of variation in CDS part average $GC3$ according to rank along genes within intron number classes (colors). Vertical bars on dots indicate standard error of means. U-shaped gradients are highly regular, and deep. Changes in CDS are especially large.

4 Intron insertion in 5' and 3' UTR

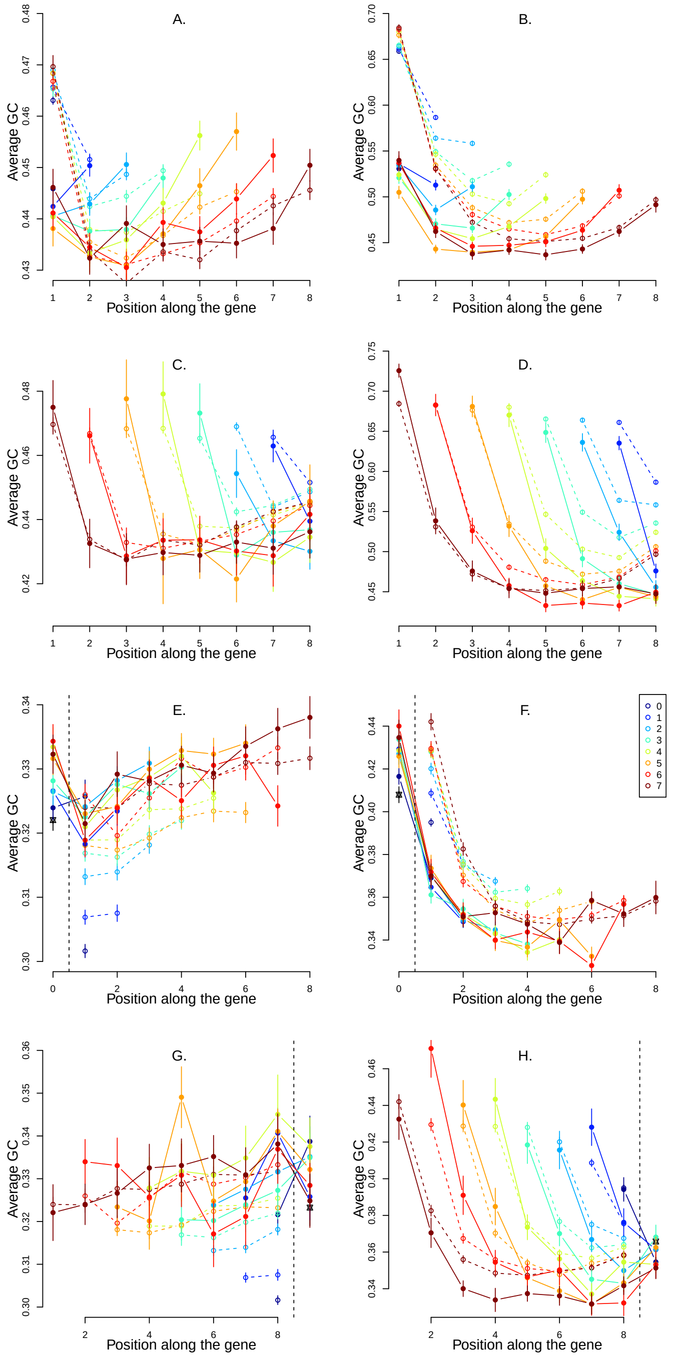

Figure S6: Comparison of CDS part and intron $G + C$ content between genes having an intron inserted within one UTR with genes having no intron inserted in UTRs. Left column: *A. thaliana*. Right column: *O. sativa*. In all panels, gradients of genes with intron inserted within UTRs (solid lines) are compared with the gradients observed in the reference set of genes having no intron inserted within UTRs (dotted lines). The colors indicate the number of

introns located in the CDS (legend shown in panel F). Bars represent standard error of means. Only gradients of genes with less than 8 introns were studied. A-D. CDS part $G + C$ gradients. A-B. Additional intron inserted within the 5'UTR. C-D. Additional intron inserted within the 3'UTR (to make the comparison easier CDS parts were aligned on their 3' end in these two last plots). In all cases, the presence of an intron located within the 5' resp. the 3' UTR decreases $G+C$ content of the 5' first resp. 3' last CDS part relatively to genes with no intron located within UTRs in both species although in *A. thaliana*, the decreases are not always significant. E-H. Intron $G + C$ gradients. E-F. Additional intron inserted within the 5'UTR. G-H. Additional intron inserted within the 3'UTR (to make the comparison easier CDS parts were aligned on their 3' end in these two last plots). In *A. thaliana* genes with less than six introns, the insertion of an intron within the 5' or the 3' UTRs leads to an increase in the $G+C$ content of all downstream introns (when the additional intron is inserted in the 5' UTR) or upstream introns (when the additional intron is inserted in the 3' UTR). In rice, the additional intron is integrated within the gradient as first (when inserted within the 5' UTR) or last (when inserted in the 3' UTR) and leads to a shift along the gradient of the downstream or the upstream introns inserted within the coding regions.

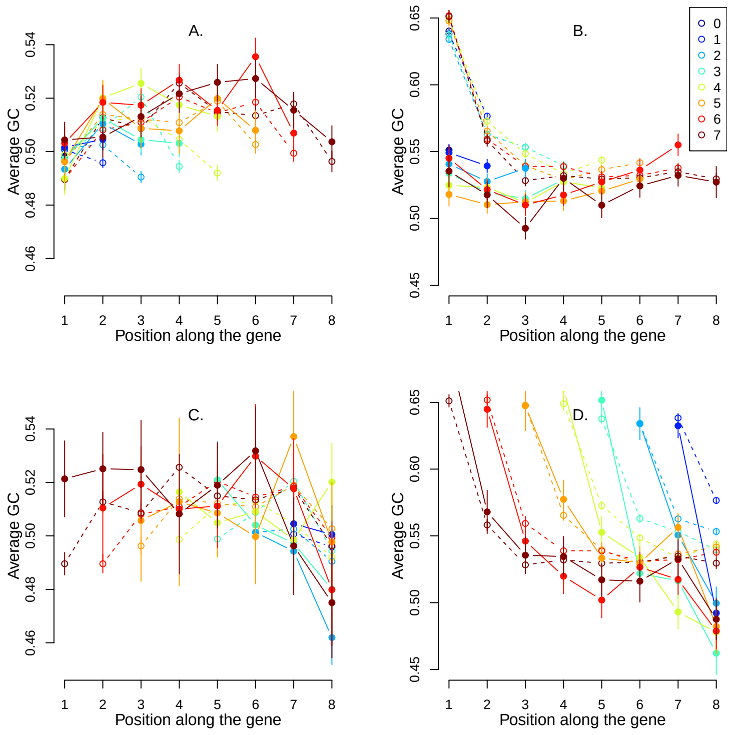

Figure S7: Comparison of CDS part $GC1$ between genes having an intron inserted within one UTR with genes having no intron inserted in UTRs. Left column: *A. thaliana*. Right column: *O. sativa*. In all panels, gradients of genes with intron inserted within UTRs (solid lines) are compared with the gradients observed in the reference set of genes having no intron inserted within UTRs (dotted lines). The colors indicate the number of introns located in the CDS (legend shown in panel B). Bars represent standard error of means. Only gradients of genes with less than 8 introns were studied. A-B. Additional intron inserted within the 5'UTR. C-D. Additional intron inserted within the 3'UTR (to make the comparison easier CDS parts were aligned on their 3' end in these two last plots).

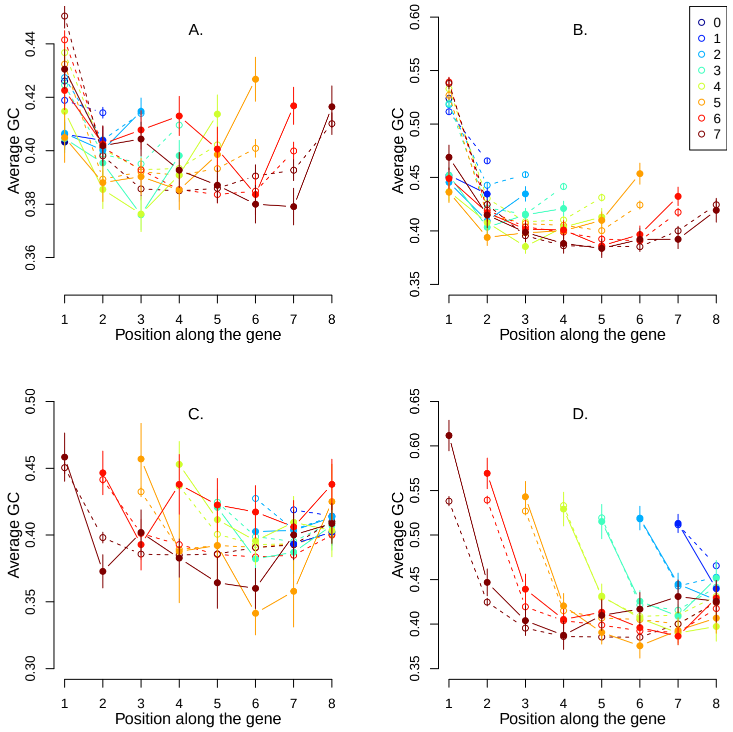

Figure S8: Comparison of CDS part $GC2$ between genes having an intron inserted within one UTR with genes having no intron inserted in UTRs. Left column: *A. thaliana*. Right column: *O. sativa*. In all panels, gradients of genes with intron inserted within UTRs (solid lines) are compared with the gradients observed in the reference set of genes having no intron inserted within UTRs (dotted lines). The colors indicate the number of introns located in the CDS (legend shown in panel B). Bars represent standard error of means. Only gradients of genes with less than 8 introns were studied. A-B. Additional intron inserted within the 5'UTR. C-D. Additional intron inserted within the 3'UTR (to make the comparison easier CDS parts were aligned on their 3' end in these two last plots).

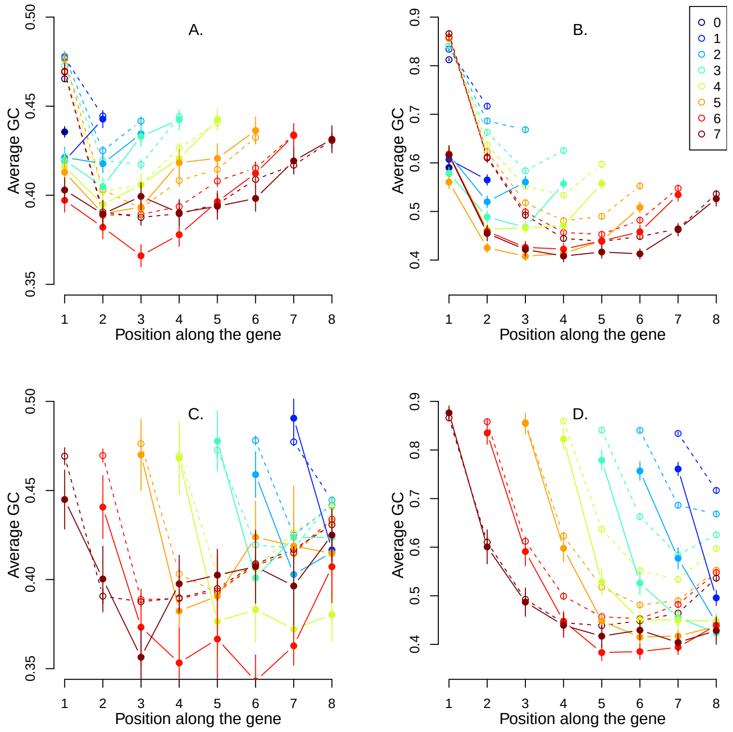

Figure S9: Comparison of CDS part *GC*3 between genes having an intron inserted within one UTR with genes having no intron inserted in UTRs. Left column: *A. thaliana*. Right column: *O. sativa*. In all panels, gradients of genes with intron inserted within UTRs (solid lines) are compared with the gradients observed in the reference set of genes having no intron inserted within UTRs (dotted lines). The colors indicate the number of introns located in the CDS (legend shown in panel B). Bars represent standard error of means. Only gradients of genes with less than 8 introns were studied. A-B. Additional intron inserted within the 5'UTR. C-D. Additional intron inserted within the 3'UTR (to make the comparison easier CDS parts were aligned on their 3' end in these two last plots).

Intron	*GC*	t	df	pval	bonferroni
0	$G+C$	10	630	$< 10^{-4}$***	***
0	$GC1$	−0.37	570	0.71	ns
0	$GC2$	7.4	570	$< 10^{-4}$***	***
0	$GC3$	9	560	$< 10^{-4}$***	***
1	$G+C$	9.2	450	$< 10^{-4}$***	***
1	$GC1$	−0.2	390	0.84	ns
1	$GC2$	2.5	400	0.012*	ns
1	$GC3$	10	390	$< 10^{-4}$***	***
2	$G+C$	10	450	$< 10^{-4}$***	***
2	$GC1$	1.5	420	0.13	ns
2	$GC2$	3.7	420	0.00022**	**
2	$GC3$	9	420	$< 10^{-4}$***	***
3	$G+C$	8.1	450	$< 10^{-4}$***	***
3	$GC1$	0.27	420	0.79	ns
3	$GC2$	3.3	440	0.001**	*
3	$GC3$	8.1	410		***
4	$G+C$	8.2	330	$< 10^{-4}$***	***
4	$GC1$	1.3	300	0.18	ns
4	$GC2$	3.1	310	$< 10^{-4}$***	.
4	$GC3$	7.3	310	$< 10^{-4}$***	***
5	$G+C$	7.8	250	$< 10^{-4}$***	***
5	$GC1$	−0.0035	220	1	ns
5	$GC2$	2.7	210	0.0065**	ns
5	$GC3$	7	220	$< 10^{-4}$***	***
6	$G+C$	6.8	300	$< 10^{-4}$***	***
6	$GC1$	−2	300	0.051.	ns
6	$GC2$	2.4	260	0.019*	ns
6	$GC3$	9.5	300	$< 10^{-4}$***	***
7	$G+C$	5.6	300	$< 10^{-4}$***	***
7	$GC1$	−1.9	310	0.061.	ns
7	$GC2$	2.3	310	0.021*	ns
7	$GC3$	8	340	$< 10^{-4}$***	***
8	$G+C$	7.2	210	$< 10^{-4}$***	***
8	$GC1$	0.092	180	0.93	ns
8	$GC2$	3.6	180	0.00047**	*
8	$GC3$	5.7	190	$< 10^{-4}$***	***

Table S5: Welsh two-samples t test on first CDS part for genes differing by the presence of an additional intron inserted in the 5' UTR in *Arabidopsis thaliana*. Intron indicates the number of intron inserted within the coding sequences, *GC* the subset of nucleotides that are compared, *t* the test statistic, *df* the degree of freedom, pval the p-value, and bonferroni indicates the significance level after a bonferroni correction for multiple tests. Levels of significance: ns for non significant, . when the p-value is < 0.1, * when the p-value is < 0.05, ** when the p-value is $< 0.01\%$ and *** when the p-value is $< 10^{-4}\%$.

Intron	*GC*	t	df	pval	bonferroni
0	$G+C$	2	110	0.044*	ns
0	GC1	−0.76	110	0.45	ns
0	GC2	1.6	110	0.12	ns
0	GC3	2.8	110	0.0063*	ns
1	$G+C$	2.7	73	0.008**	ns
1	GC1	−0.42	69	0.67	ns
1	GC2	1.2	71	0.24	ns
1	GC3	2.4	69	0.017*	ns
2	$G+C$	3.3	52	0.002**	.
2	GC1	2.8	52	0.008**	ns
2	GC2	0.051	51	0.96	ns
2	GC3	2	51	0.046*	ns
3	$G+C$	2	39	0.058.	ns
3	GC1	0.84	37	0.41	ns
3	GC2	0.13	37	0.9	ns
3	GC3	1.7	40	0.098.	ns
4	$G+C$	1.3	32	0.2	ns
4	GC1	−1.9	32	0.067.	ns
4	GC2	−0.058	31	0.95	ns
4	GC3	4	32	0.00035**	*
5	$G+C$	−0.037	17	0.97	ns
5	GC1	0.22	17	0.83	ns
5	GC2	−0.85	16	0.41	ns
5	GC3	0.72	17	0.48	ns
6	$G+C$	0.32	26	0.75	ns
6	GC1	0.93	25	0.36	ns
6	GC2	−2	25	0.059.	ns
6	GC3	1.3	25	0.21	ns
7	$G+C$	1.1	31	0.28	ns
7	GC1	1	30	0.32	ns
7	GC2	0.099	32	0.92	ns
7	GC3	0.38	33	0.71	ns
8	$G+C$	0.89	28	0.38	ns
8	GC1	1.7	27	0.094.	ns
8	GC2	−0.84	26	0.41	ns
8	GC3	0.52	29	0.61	ns

Table S6: Welsh two-samples t test on last CDS part for genes differing by the presence of an additional intron inserted in the 3' UTR in *Arabidopsis thaliana*. (see table S5 caption).

Intron	*GC*	t	df	pval	bonferroni
0	G+C	10	630	$< 10^{-4}$***	***
0	GC1	−0.37	570	0.71	ns
0	GC2	7.4	570	$< 10^{-4}$***	***
0	GC3	9	560	$< 10^{-4}$***	***
1	G+C	0.51	450	0.61	ns
1	GC1	−2	410	0.044*	ns
1	GC2	1.9	380	0.055*	ns
1	GC3	0.33	390	0.74	ns
2	G+C	−0.76	440	0.45	ns
2	GC1	−2.7	470	0.0067**	ns
2	GC2	−0.15	420	0.88	ns
2	GC3	1.4	450	0.17	ns
3	G+C	0.55	420	0.59	ns
3	GC1	−1.5	390	0.12	ns
3	GC2	1.8	390	0.068.	ns
3	GC3	0.3	410	0.76	ns
4	G+C	−3.6	320	0.00038**	*
4	GC1	−3.4	320	0.00076**	*
4	GC2	−1.5	280	0.14	ns
4	GC3	−0.27	310	0.79	ns
5	G+C	−3	220	0.0035**	ns
5	GC1	−0.66	220	0.51	ns
5	GC2	−2.9	210	0.0038**	ns
5	GC3	−0.47	210	0.64	ns
6	G+C	−2.2	260	0.029*	ns
6	GC1	−1.1	260	0.29	ns
6	GC2	−2.2	260	0.029*	ns
6	GC3	0.048	270	0.96	ns
7	G+C	−1.3	300	0.18	ns
7	GC1	−1	320	0.31	ns
7	GC2	−0.71	260	0.48	ns
7	GC3	−0.079	280	0.94	ns
8	G+C	−1.9	180	0.059	ns
8	GC1	1	200	0.31	ns
8	GC2	−1.8	190	0.069.	ns
8	GC3	−1.6	170	0.12	ns

Table S7: Within intron number class Welsh two-samples test on last CDS parts $G + C$ content and codon position $G + C$ content of genes with and without an intron inserted in their 5' UTR in *Arabidopsis thaliana*. Intron indicates the number of intron inserted within the coding sequences, *GC* the subset of nucleotides that are compared, *t* the test statistic, *df* the degree of freedom, pval the p-value, and bonferroni indicate the significance level after a bonferroni correction for multiple tests (see table S5 caption).

Intron	*GC*	t	df	pval	bonferroni
0	G+C	2	110	0.044*	ns
0	GC1	−0.76	110	0.45	ns
0	GC2	1.6	110	0.12	ns
0	GC3	2.8	110	0.0063**	ns
1	G+C	0.53	72	0.59	ns
1	GC1	−0.37	69	0.71	ns
1	GC2	2.7	71	0.0095**	ns
1	GC3	−1.2	70	0.23	ns
2	G+C	1.9	51	0.058	ns
2	GC1	0.0018	51	1	ns
2	GC2	1.6	50	0.11	ns
2	GC3	1.5	52	0.15	ns
3	G+C	−0.85	38	0.4	ns
3	GC1	−1.5	38	0.14	ns
3	GC2	0.28	39	0.78	ns
3	GC3	−0.3	38	0.77	ns
4	G+C	−1.1	31	0.3	ns
4	GC1	−1	31	0.32	ns
4	GC2	−0.94	32	0.36	ns
4	GC3	0.069	31	0.95	ns
5	G+C	−0.76	17	0.46	ns
5	GC1	−0.41	17	0.68	ns
5	GC2	−0.91	17	0.38	ns
5	GC3	0.31	17	0.76	ns
6	G+C	0.077	26	0.94	ns
6	GC1	−1	25	0.31	ns
6	GC2	−0.3	26	0.76	ns
6	GC3	1.6	26	0.12	ns
7	G+C	−0.62	32	0.54	ns
7	GC1	−2.1	33	0.04*	ns
7	GC2	−0.43	32	0.67	ns
7	GC3	1.4	33	0.17	ns
8	G+C	−1.8	29	0.086.	ns
8	GC1	−0.25	27	0.8	ns
8	GC2	−2.9	29	0.0064**	ns
8	GC3	0.7	28	0.49	ns

Table S8: Within intron number class Welsh two-samples test on first CDS parts $G+C$ content and codon position $G+C$ content of genes with and without an intron inserted in their 3' UTR in *Arabidopsis thaliana*. Intron indicates the number of intron inserted within the coding sequences, *GC* the subset of nucleotides that are compared, *t* the test statistic, *df* the degree of freedom, pval the p-value, and bonferroni indicate the significance level after a bonferroni correction for multiple tests (see table S5 caption).

Intron	*GC*	t	df	pval	bonferroni
0	G+C	27	580	$< 10^{-4}$	***
0	GC1	21	600	$< 10^{-4}$	***
0	GC2	17	630	$< 10^{-4}$	***
0	GC3	25	580	$< 10^{-4}$	***
1	G+C	21	390	$< 10^{-4}$	***
1	GC1	14	420	$< 10^{-4}$	***
1	GC2	9.4	420	$< 10^{-4}$	***
1	GC3	20	390	$< 10^{-4}$	***
2	G+C	18	300	$< 10^{-4}$	***
2	GC1	12	310	$< 10^{-4}$	***
2	GC2	8.9	320	$< 10^{-4}$	***
2	GC3	16	290	$< 10^{-4}$	***
3	G+C	21	340	$< 10^{-4}$	***
3	GC1	12	340	$< 10^{-4}$	***
3	GC2	8	360	$< 10^{-4}$	***
3	GC3	19	310	$< 10^{-4}$	***
4	G+C	21	280	$< 10^{-4}$	***
4	GC1	12	270	$< 10^{-4}$	***
4	GC2	9.8	290	$< 10^{-4}$	***
4	GC3	15	250	$< 10^{-4}$	***
5	G+C	23	290	$< 10^{-4}$	***
5	GC1	14	280	$< 10^{-4}$	***
5	GC2	8.5	280	$< 10^{-4}$	***
5	GC3	20	250	$< 10^{-4}$	***
6	G+C	17	210	$< 10^{-4}$	***
6	GC1	11	230	$< 10^{-4}$	***
6	GC2	8.4	230	$< 10^{-4}$	***
6	GC3	14	190	$< 10^{-4}$	***
7	G+C	14	160	$< 10^{-4}$	***
7	GC1	9.1	160	$< 10^{-4}$	***
7	GC2	5.5	180	$< 10^{-4}$	***
7	GC3	13	140	$< 10^{-4}$	***
8	G+C	16	150	$< 10^{-4}$	***
8	GC1	9.7	150	$< 10^{-4}$	***
8	GC2	8	160	$< 10^{-4}$	***
8	GC3	13	140	$< 10^{-4}$	***

Table S9: Within intron number class Welsh two-samples test on first CDS parts $G + C$ content and codon position $G + C$ content of genes with and without an intron inserted in their 5' UTR in *Oryza sativa.* Intron indicates the number of intron inserted within the coding sequences, *GC* the subset of nucleotides that are compared, *t* the test statistic, *df* the degree of freedom, pval the p-value, and bonferroni indicate the significance level after a bonferroni correction for multiple tests (see table S5 caption).

Intron	*GC*	t	df	pval	bonferroni
0	G+C	5.6	300	$< 10^{-4}$***	***
0	GC1	2.6	300	0.0097**	ns
0	GC2	0.84	290	0.4	ns
0	GC3	7.7	300	$< 10^{-4}$***	***
1	G+C	12	180	$< 10^{-4}$***	***
1	GC1	7.2	170	$< 10^{-4}$***	***
1	GC2	2.2	170	0.031*	ns
1	GC3	14	190	$< 10^{-4}$***	***
2	G+C	12	120	$< 10^{-4}$***	***
2	GC1	4.3	110	$< 10^{-4}$***	**
2	GC2	2	110	0.045*	ns
2	GC3	12	120	$< 10^{-4}$***	***
3	G+C	10	90	$< 10^{-4}$***	***
3	GC1	4.8	76	$< 10^{-4}$***	**
3	GC2	−0.49	74	0.62	ns
3	GC3	12	94	$< 10^{-4}$***	***
4	G+C	8.6	77	$< 10^{-4}$***	***
4	GC1	4	69	0.00016**	**
4	GC2	2	69	0.048*	ns
4	GC3	9.9	95	$< 10^{-4}$***	***
5	G+C	6.7	72	$< 10^{-4}$***	***
5	GC1	2.9	63	0.0051**	ns
5	GC2	1	65	0.32	ns
5	GC3	6.7	78	$< 10^{-4}$***	***
6	G+C	7.1	85	$< 10^{-4}$***	***
6	GC1	4.2	67	$< 10^{-4}$***	**
6	GC2	−0.75	66	0.46	ns
6	GC3	6.8	85	$< 10^{-4}$***	***
7	G+C	4	47	0.0002**	**
7	GC1	2.7	47	0.01*	ns
7	GC2	−0.047	44	0.96	ns
7	GC3	3.7	46	0.00056**	*
8	G+C	8.2	80	$< 10^{-4}$***	***
8	GC1	3	64	0.0035*	ns
8	GC2	2.2	66	0.028*	ns
8	GC3	6.5	81	$< 10^{-4}$***	***

Table S10: Within intron number class Welsh two-samples test on last CDS parts $G + C$ content and codon position $G + C$ content of genes with and without an intron inserted in their 3' UTR in *Oryza sativa.* Intron indicates the number of intron inserted within the coding sequences, *GC* the subset of nucleotides that are compared, *t* the test statistic, *df* the degree of freedom, pval the p-value, and bonferroni indicate the significance level after a bonferroni correction for multiple tests (see table S5 caption).

Intron	*GC*	t	df	pval	bonferroni
0	G+C	27	580	$< 10^{-4}$***	***
0	GC1	21	600	$< 10^{-4}$***	***
0	GC2	17	630	$< 10^{-4}$***	***
0	GC3	25	580	$< 10^{-4}$***	***
1	G+C	12	450	$< 10^{-4}$***	***
1	GC1	6.9	480	$< 10^{-4}$***	***
1	GC2	5.1	440	$< 10^{-4}$***	***
1	GC3	13	460	$< 10^{-4}$***	***
2	G+C	7.7	370	$< 10^{-4}$***	***
2	GC1	2.2	330	0.029*	ns
2	GC2	2.5	320	0.013*	ns
2	GC3	8.3	360	$< 10^{-4}$***	***
3	G+C	5.3	400	$< 10^{-4}$***	***
3	GC1	1.5	370	0.14	ns
3	GC2	2.8	360	0.006**	ns
3	GC3	5.4	420	$< 10^{-4}$***	***
4	G+C	4.3	350	$< 10^{-4}$***	**
4	GC1	2.7	300	0.0073**	ns
4	GC2	2.1	290	0.035*	ns
4	GC3	3	350	0.003**	ns
5	G+C	1.5	320	0.15	ns
5	GC1	1.6	300	0.12	ns
5	GC2	−2.8	260	0.006**	ns
5	GC3	3.4	320	0.00087**	*
6	G+C	−0.88	240	0.38	ns
6	GC1	−1.9	220	0.053.	ns
6	GC2	−1.6	230	0.12	ns
6	GC3	0.9	250	0.37	ns
7	G+C	0.62	160	0.54	ns
7	GC1	0.19	150	0.85	ns
7	GC2	0.43	170	0.67	ns
7	GC3	0.61	180	0.54	ns
8	G+C	−0.71	150	0.48	ns
8	GC1	1.7	150	0.09.	ns
8	GC2	−3	150	0.0029**	ns
8	GC3	−0.038	160	0.97	ns

Table S11: Within intron number class Welsh two-samples test on last CDS parts $G + C$ content and codon position $G + C$ content of genes with and without an intron inserted in their 5' UTR in *Oryza sativa.* Intron indicates the number of intron inserted within the coding sequences, *GC* the subset of nucleotides that are compared, *t* the test statistic, *df* the degree of freedom, pval the p-value, and bonferroni indicate the significance level after a bonferroni correction for multiple tests (see table S5 caption).

Intron	*GC*	t	df	pval	bonferroni
0	G+C	5.6	300	$< 10^{-4}$***	***
0	GC1	2.6	300	0.0097**	ns
0	GC2	0.84	290	0.4	ns
0	GC3	7.7	300	$< 10^{-4}$***	***
1	G+C	3	170	0.0031**	ns
1	GC1	0.62	170	0.53	ns
1	GC2	−0.15	170	0.88	ns
1	GC3	5.1	180	$< 10^{-4}$***	***
2	G+C	2.5	110	0.015*	ns
2	GC1	−0.0021	110	1	ns
2	GC2	−0.064	110	0.95	ns
2	GC3	4.3	110	$< 10^{-4}$***	**
3	G+C	1.3	77	0.2	ns
3	GC1	−1.1	82	0.26	ns
3	GC2	0.21	76	0.83	ns
3	GC3	2.9	77	0.0052**	ns
4	G+C	0.67	68	0.5	ns
4	GC1	−0.69	70	0.49	ns
4	GC2	0.2	69	0.84	ns
4	GC3	1.6	69	0.12	ns
5	G+C	−0.34	66	0.73	ns
5	GC1	0.017	64	0.99	ns
5	GC2	−0.9	67	0.37	ns
5	GC3	0.048	67	0.96	ns
6	G+C	−0.0098	66	0.99	ns
6	GC1	0.49	70	0.62	ns
6	GC2	−1.7	68	0.097.	ns
6	GC3	0.98	66	0.33	ns
7	G+C	−4.6	57	$< 10^{-4}$***	**
7	GC1	−3.2	57	0.002**	.
7	GC2	−4.1	49	0.00017**	**
7	GC3	−0.68	54	0.5	ns
8	G+C	0.52	66	0.61	ns
8	GC1	0.6	64	0.55	ns
8	GC2	−0.22	67	0.83	ns
8	GC3	0.63	65	0.53	ns

Table S12: Within intron number class Welsh two-samples test on first CDS parts $G + C$ content and codon position $G + C$ content of genes with and without an intron inserted in their 3' UTR in *Oryza sativa.* Intron indicates the number of intron inserted within the coding sequences, *GC* the subset of nucleotides that are compared, *t* the test statistic, *df* the degree of freedom, pval the p-value, and bonferroni indicate the significance level after a bonferroni correction for multiple tests (see table S5 caption).

5 Comparisons between pairs of related genes

$G+C$	No intron	5' intron	t'	df	pval
First CDS $GC1$	0.48	0.50	−2.75	369	0.0055
First CDS $GC2$	0.39	0.39	1.22	369	0.2153
First CDS $GC3$	0.44	0.42	2.15	369	0.0323
First CDS $G+C$	0.44	0.44	0.51	369	0.6133
First intron $G+C$	0.32	0.32	0.51	369	0.6133
$G+C$	No intron	3' intron	t'	df	pval
Last CDS $GC1$	0.46	0.46	0.73	185	0.4652
Last CDS $GC2$	0.40	0.42	−1.90	185	0.0596
Last CDS $GC3$	0.41	0.41	0.03	185	0.9745
Last CDS $G+C$	0.42	0.43	−1.59	185	0.1134
Last intron $G+C$	0.31	0.32	−2.12	117	0.0365

Table S13: Comparisons between external CDS part and intron average $G+C$ content between pairs of related genes having no intron in UTRs or having an intron inserted within the 5'UTR in *A. thaliana*. $G+C$ column: type of sequences and or subset of sequences compared; CDS part first (GC1), second (GC2) or third position (GC2) within codons and average over all position ($G+C$). For introns, first or last intron inserted in the CDS $G+C$ (second and penultimate introns). Each subsample means are indicated in the dataset named columns. Paired Welsh two samples t-test statistics (t'), degrees of freedom (df) and p-values (p).

$G+C$	No intron	5' intron	t'	df	pval
First CDS $GC1$	0.63	0.59	3.80	133	0.0002
First CDS $GC2$	0.49	0.47	2.02	133	0.0450
First CDS $GC3$	0.83	0.69	7.54	133	$< 10^{-4}$
First CDS $G+C$	0.65	0.58	7.10	133	$< 10^{-4}$
First intron $G+C$	0.41	0.38	4	109	0.0001
$G+C$	No intron	3' intron	t'	df	pval
Last CDS $GC1$	0.53	0.53	−0.28	41	0.7794
Last CDS $GC2$	0.46	0.45	0.3	41	0.7691
Last CDS $GC3$	0.57	0.55	0.97	41	0.3377
Last CDS $G+C$	0.55	0.51	0.64	41	0.5253
Last intron $G+C$	0.36	0.35	260	42	0.3766

Table S14: Comparisons between external CDS parts and introns average $G+C$ content between pairs of related genes having no intron in UTRs or having an intron inserted within the 5'UTR in *O. sativa*. $G+C$ column: type of sequences and or subset of sequences compared; CDS part first (GC1), second (GC2) or third position (GC2) within codons and average over all position ($G+C$). For introns, first or last intron inserted in the CDS $G+C$ (second and penultimate introns). Each subsample means are indicated in the dataset named columns. Paired Welsh two samples t-test statistics (t'), degrees of freedom (df) and p-values (p).

6 CDS part $G+C$ content distributions according to first intron length

As already noticed in a number of species including rice and *A. thaliana*, first introns tend to be longer in average than introns of all higher ranks. In fact in the two plant species, intron lengths tend to be either short around 90 nts in both species, or longer, above 149 nts in *A. thaliana* and 245 nts in rice, the two intron groups being observed in all intron ranks along genes (fig. S9F in rice,

S8F in *A. thaliana*). The increase in average first intron length is due to both a higher proportion of long introns in first introns compared to other ranks and a larger size of the long introns.

The huge variation in first intron length is associated with large changes in coding regions $G+C$ content in rice and negligible changes in *A. thaliana*. In rice, CDS part rank position but also gene intron number have a dramatic impact on CDS part $G+C$ content (and the length of the first intron affects both CDS part and intron $G+C$ content). As shown in fig. S9, when first introns are short (below 245 nt long), the second CDS part tend to be $G+C$-rich, while when first intron are long (above 245 nt long), the second CDS part $G+C$ content tend to be $G+C$-poor. As a result, when both groups of genes are pooled, a distinct bimodality in second CDS part $G+C$ content distribution is observed in all intron number classes. For low intron number genes, this effect of first intron length lasts over all downstream CDS parts. In higher intron number genes, internal CDS part $G+C$ content distribution become progressively unimodal as one progresses towards the central regions of the genes. Therefore in rice, the smoothness of the U-shaped gradients is at least partially due to the variation in the number of genes displaying a long first intron. Unlike in rice, *A. thaliana* CDS part $G+C$ content distributions are all unimodal and changes with rank position along the genes mainly result from shift of the modes of the distribution (fig. S8).

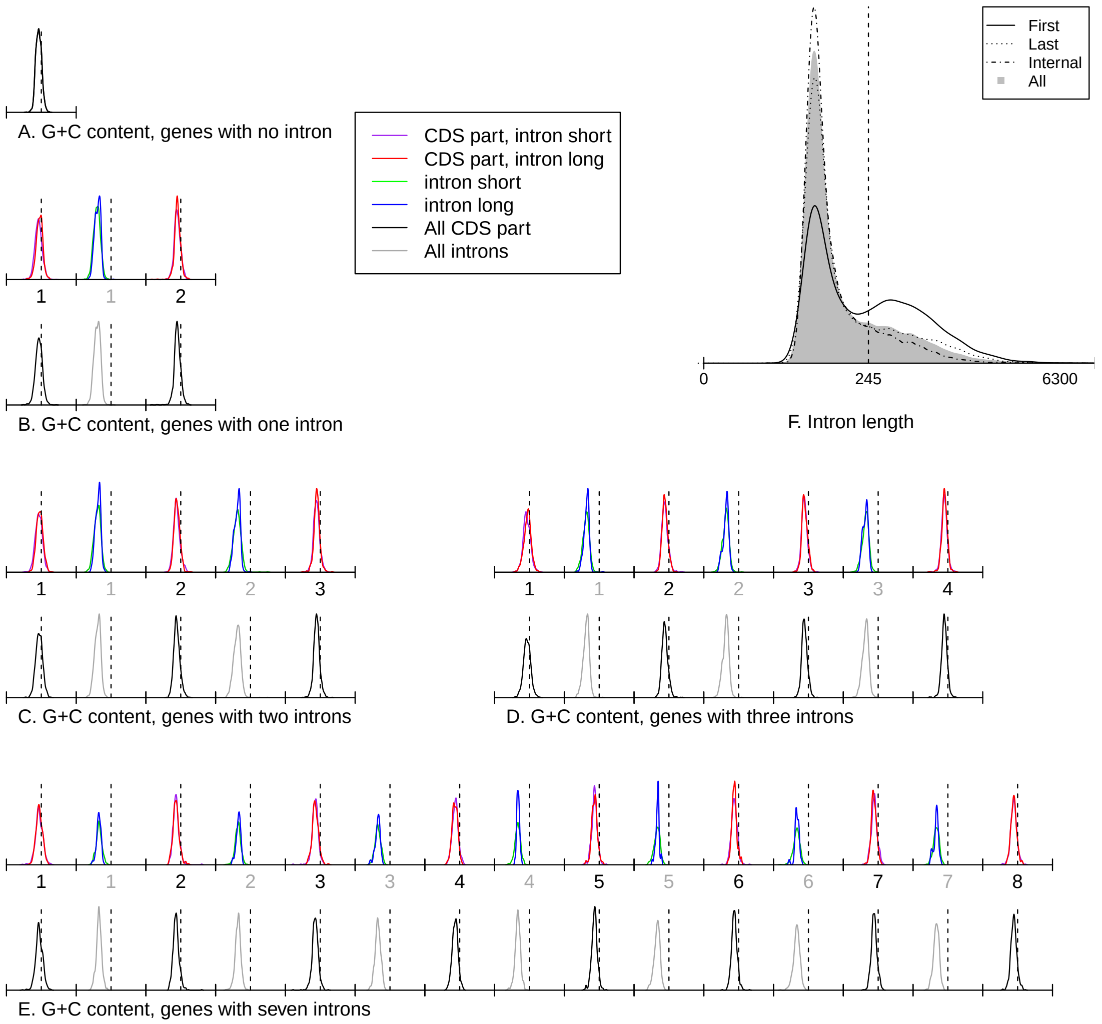

Figure S10: CDS part and intron $G+C$ content variation in *A. thaliana*. A. CDS $G+C$ content density outline of intronless genes. For intronic genes(B-E), the densities of each CDS part and intron $G+C$ content are plotted in two rows. The first row corresponds to CDS part $G+C$ content distributions in two categories of genes, genes having either a short or a long first intron (short introns are below 245 nucleotides, long introns above 245 nts). The second row corresponds to the overall distributions when the two former categories are pooled (CDS parts of all genes within an intron number class). Each row is formed by the alternance of CDS part and intron densities, colors of the density outlines being indicated in the legend in the plot. The density plots of each CDS part or intron have the same distribution supports between 0 and 1, the limits being indicated by the ticks on the axes below the density outlines, the dashed vertical lines indicating a $G+C$ content of 0.5. The digits plotted below each upper row indicate the CDS part (in black) or intron rank (in grey) along genes. B, C, D and E: Genes with one intron, two, three and seven introns respectively. F. Patterns of variation in intron length. The support of the distribution is in logarithmic scale, the dotted line is placed at 245 nts. The grey area shows length distribution pooling all introns, the plain line outlines the density of first intron length, the dotted line outlines the density of last intron length, and the dashed-dotted line the outlines the density of internal intron length.

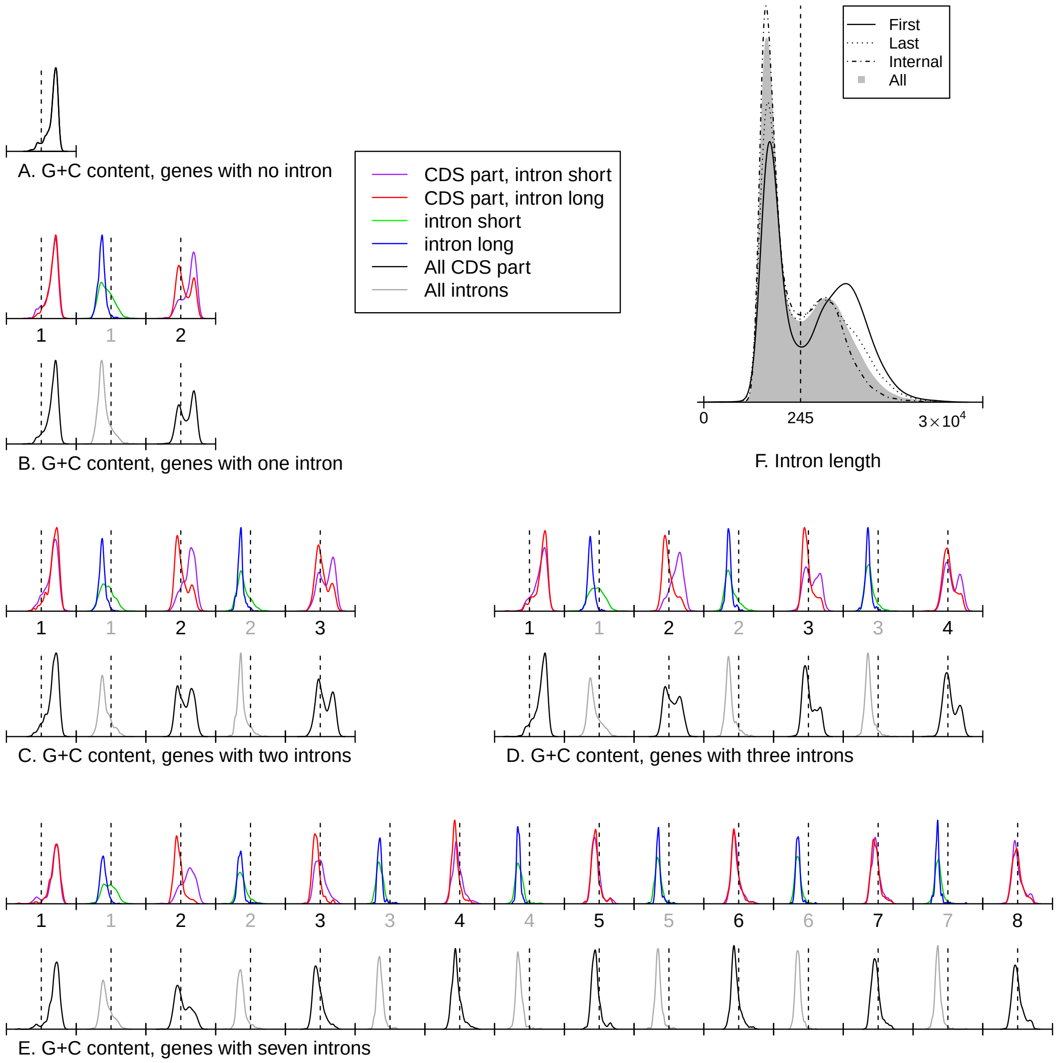

Figure S11: CDS part and intron $G+C$ content variation in *O. sativa*. A. CDS $G+C$ content density outline of intronless genes. For intronic genes(B-E), the densities of each CDS part and intron $G+C$ content are plotted in two rows. The first row corresponds to CDS part $G+C$ content distributions in two categories of genes, genes having either a short or a long first intron (short introns are below 245 nucleotides, long introns above 245 nts). The second row corresponds to the overall distributions when the two former categories are pooled (CDS parts of all genes within an intron number class). Each row is formed by the alternance of CDS part and intron densities, colors of the density outlines being indicated in the legend in the plot. The density plots of each CDS part or intron have the same distribution supports between 0 and 1, the limits being indicated by the ticks on the axes below the density outlines, the dashed vertical lines indicating a $G+C$ content of 0.5. The digits plotted below each upper row indicate the CDS part (in black) or intron rank (in grey) along genes. B, C, D and E: Genes with one intron, two, three and seven introns respectively. F. Patterns of variation in intron length. The support of the distribution is in logarithmic scale, the dotted line is placed at 245 nts. The grey area shows length distribution pooling all introns, the plain line outlines the density of first intron length, the dotted line outlines the density of last intron length, and the dashed-dotted line the outlines the density of internal intron length.

7 Contributions to CDS $G + C$ content variances

If codon positions are independent, the expected variance of CDS parts is equal to

$$\hat{\sigma}^2(GC_{ij}^{CDS}) = \frac{1}{9}(\sigma^2(GC1_{ij}^{CDS}) + \sigma^2(GC2_{ij}^{CDS}) + \sigma^2(GC3_{ij}^{CDS})) \tag{1}$$

In *A. thaliana*, observed CDS part GC-content variances are almost always lower than the one expected when codon positions are independent (Figure S12A). In *O. sativa*, the observed CDS part GC-content variances are lower than expected in internal CDS parts of genes with high intron numbers while they are higher in genes with low intron number or external CDS parts (Figure S12B).

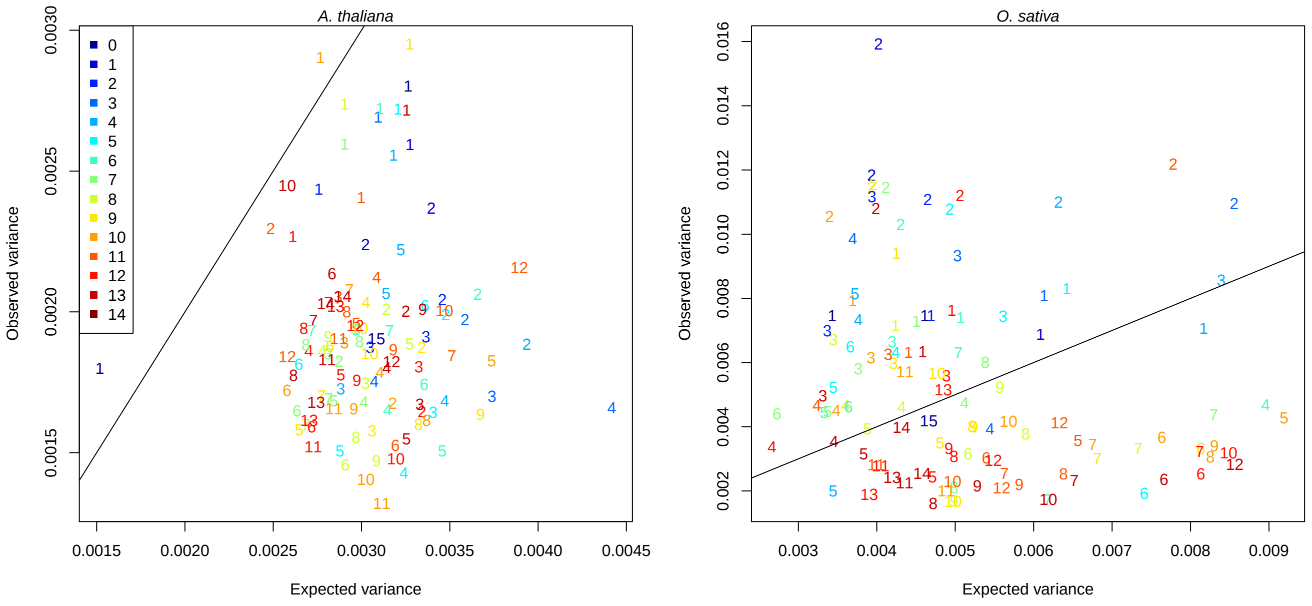

Figure S12: Expected *versus* observed variance in CDS part GC-content in *A. thaliana* (left) and *O. sativa* (right). The colors indicate gene intron number (legend in the left panel) and the numbers the rank of the CDS parts. The line indicates equal expected and observed variances.

To further described variance pattern at CDS scale, for each intron number class i, CDS $G + C$ content variance ($\sigma^2(GC_i^{CDS})$) were re-arranged into three components:

$$\sigma^2(GC_i^{CDS}) = \underbrace{\sum_{j=1}^{j=i+1} \sigma^2(V_{ij}) + \sum_{j=1}^{j=i+1} \sigma^2(F_{ij})}_{Var\ intra} + \underbrace{2\sum_{j=1}^{j=i}\sum_{k=j+1}^{k=i+1} \sigma(V_{ij}, V_{ik}) + 2\sum_{j=1}^{j=i}\sum_{k=j+1}^{k=i+1} \sigma(F_{ij}, F_{ik})}_{Cov\ inter}$$
$$+ \underbrace{2\sum_{j=1}^{j=i+1}\sum_{k=1}^{k=i+1} \sigma(V_{ij}, F_{ik})}_{Cov\ VF} \tag{2}$$

where j and k indicate the rank of the CDS part and V_{ij} and F_{ij} are the respective contributions of synonymous and non-synonymous nucleotides to GC-content of the j-th CDS part. Variation

in each of the three components, the sum of the CDS part variances (Var intra), the sum of the covariances among CDS parts (Cov inter) and the sum of the covariances among synonymous and non-synonymous nucleotides within CDS parts (Cov VF) are shown in table S15 for *A. thaliana* and S16 for rice.

Intron	Var intra	Cov inter	Cov VF	Var CDS	Cov VF contribution
0	0.002	0	−0.00019	0.0018	−9.7%
1	0.014	−0.013	−0.00017	0.0014	−11%
2	0.013	−0.011	−0.00011	0.0011	−9.1%
3	0.011	−0.01	−0.00025	0.00075	−25%
4	0.0086	−0.0077	−0.00023	0.00068	−25%
5	0.0083	−0.0075	−0.00024	0.00059	−29%
6	0.0073	−0.0066	−0.00022	0.00052	−30%
7	0.0063	−0.0057	−0.0002	0.00041	−33%
8	0.0056	−0.005	−0.00019	0.00044	−31%
9	0.0047	−0.0042	−0.0002	0.00035	−36%
10	0.0046	−0.004	−0.00024	0.00031	−44%
11	0.0038	−0.0032	−0.0002	0.00039	−34%
12	0.0043	−0.0038	−0.00018	0.0003	−37%
13	0.0037	−0.0033	−0.00015	0.0003	−33%
14	0.0034	−0.0028	−0.00025	0.0003	−46%

Table S15: CDS GC-content variance components in *A. thaliana*. Gene intron number is indicated in the first column (Intron), the GC-content CDS variance (Var CDS) being equal to the sum of CDS part variances per rank (Var intra), the sum of the covariances among rank (Cov inter) and the sum of covariances between variable and fixed nucleotide within CDS parts (Cov VF). The first two components are large but almost completely anti-correlated. Most of the decrease in total variance is due to the variations in these components. The sums of the covariances among synonymous and non-synonymous nucleotides are always negatives and decrease as intron number increases. Although small compared to the two others, it further accounts for a decrease of the total variance that ranges between 9 and 46% (Cov VF contribution, computed as percentage of Cov VF in the sum of the two first components).

intron	Var intra	Cov inter	Cov VF	Var CDS	Cov VF contribution
0	0.0056	0	0.0019	0.0075	+33%
1	0.033	−0.028	0.0016	0.0066	+33%
2	0.027	−0.023	0.0015	0.0062	+33%
3	0.021	−0.017	0.001	0.0053	+24%
4	0.016	−0.012	0.00091	0.0048	+23%
5	0.014	−0.011	0.00075	0.0043	+21%
6	0.012	−0.0092	0.0005	0.0034	+17%
7	0.01	−0.0074	0.00048	0.0031	+18%
8	0.0088	−0.0067	0.00026	0.0023	+13%
9	0.0079	−0.0057	0.00014	0.0024	+6.3%
10	0.0066	−0.0047	0.0001	0.002	+5.6%
11	0.0059	−0.0043	0.000005	0.0015	+0.34%
12	0.006	−0.0045	0.00003	0.0015	+1.7%
13	0.0045	−0.0034	0.00007	0.0012	+6.1%
14	0.0049	−0.0039	0.0001	0.0011	+9.2%

Table S16: CDS $G+C$ content variance components in *Oryza sativa.* Gene intron number is indicated in the first column (Intron), the $G + C$ content CDS variance (Var CDS) being equal to the sum of CDS part variances per rank (Var intra), the sum of the covariances among rank (Cov inter) and the sum of covariances between variable and fixed nucleotide within CDS parts (Cov VF). The first two components are large but almost completely anti-correlated. Most of the decrease in total variance is due to the variations in these components. The sums of the covariances among among synonymous and non-synonymous nucleotides are always positives and decrease as intron number increases. Although small compared to the two others (Cov VF contribution, computed as percentage of Cov VF in the sum of the two first components), it further accounts for an increase of the total variance that can reach 33% for genes with low intron number. It vanishes as intron number increases, contributing to a reduction of the total variance.
